# Cation hydration by confined water and framework-atoms have crucial role on thermodynamics of clay swell﻿ing

**DOI:** 10.1038/s41598-022-21349-3

**Published:** 2022-10-24

**Authors:** Sai Adapa, Ateeque Malani

**Affiliations:** 1 Department of Chemical Engineering, Indian Institute of Technology Bombay, Mumbai, 400076 India; 2 Research and Development Division, Tata Steel Limited, Jamshedpur, 831001 India

**Keywords:** Geochemistry, Chemical engineering, Physical chemistry, Surface chemistry, Theoretical chemistry

## Abstract

The swelling capacity and stability of clay play a crucial role in various areas ranging from cosmetics to oil extraction; hence change in their swelling behaviour after cation exchange with the surrounding medium is important for their efficient utilisation. Here we focus on understanding the role of different hydration properties of cation on the thermodynamics of clay swelling by water adsorption. We have used mica as the reference clay, Na$^+$, Li$^+$, and H$^+$ ions as the interstitial cations, and performed grand canonical Monte Carlo simulations of water adsorption in mica pores (of widths $d = 4-40$ Å). The disjoining pressure ($\Pi$), swelling free energy ($\Delta \Omega ^{ex}$), and several structural properties of confined water and ions were calculated to perform a thermodynamic analysis of the system. We expected higher water density in H-mica pores ($\rho_{ \hbox{H}}$) due to the smaller size of $\hbox {H}^+$ ions having higher hydration energy. However, the counter-intuitive trend of $\rho _{\hbox{Li}}> \rho _{\hbox{Na}} > \rho_b$ (bulk density) $> \rho_{\hbox{H}}$ was observed due to adsorption energy, where the interaction of water with mica framework atoms was also found to be significant. All three mica systems exhibited oscillatory behaviour in the $\Pi$ and $\Delta \Omega ^{ex}$ profiles, diminishing to zero for $d \ge 11$ Å. The $\Delta \Omega ^{ex}$ for Na-mica is characterised by global minima at $d=6 {\hbox {\AA}}$   corresponding to crystalline swelling with significant and multiple barriers for crystalline swelling to osmotic swelling ($d > 12$ Å). A shift in the location of global minima of $\Delta \Omega ^{ex}$ towards the higher *d* values and $\Delta \Omega ^{ex}$ becoming more repulsive is observed in the increasing order of hydration energy of $\hbox {Na}^+$, $\hbox {Li}^+$, and $\hbox {H}^+$ ions. The $\Delta \Omega ^{ex} > 0$ for all *d* in the H-mica system thus favours osmotic swelling. We found that the Na$^+$ ions hydrate more surface oxygens, act as anchors, and hold the mica pore together (at smaller *d*), by sharing hydrating water with ions of the opposite side, forming an electrostatically connected mica-Na-water-Na-mica bridge. The Li$^+$ ions do hydrate surface oxygen atoms, albeit in lesser numbers, and sharing of hydration shell with nearby Li$^+$ ions is also minimum. Hydration by surface atoms and water sharing, both, are minimum in the H$^+$ ion case, as they are mostly present in the center of the pore as diffusive ions, thus exerting a consistent osmotic pressure on the mica frameworks, favouring swelling.

## Introduction

Traditionally, clay swelling has been investigated due to its vital role in oil and gas extraction. During drilling and extraction of oil, generally water based fluid is used to lubricate the drill, carry rock cutting and displace oil. However, the drilling fluid intercalates nearby rock and clay leading to swelling, which may reduce oil production and collapse of well. To prevent these technical problems and design suitable inhibitors, understanding fundamentals of clay swelling is important. Recently, clay swelling has found a renewed interest due to wide reach of importance of clays in $\hbox {CO}_{\hbox {2}}$ sequestration^1^, pharmaceuticals^2^, cosmetics^3^, disposal of radioactive waste^4,5^, nutrient supply in soil^6^, cement^7^, paint^8^, catalysis^9^ and many other industries. Clays are layered materials made of tetrahedral and octahedral sheets with isomorphic substitution in these layers which create charge imbalance, compensated by the loose, often hydrated, interstitial cations. The composition of clays (both, framework atoms and cations) vary locally at platelets level (in nm to micron range) and globally (at cm to km range) due to geological process, geographical location and climate. Further, clays could have different interstitial cation due to ion exchange with the surrounding medium occurring at various time scales. Understanding the role of cation properties on clay-swelling is important to design appropriate inhibitors and promoters. Here we investigate the role of cation hydration properties (hydration energies, hydration size, solvation by water and surface atoms, and coordination number) on swelling pressure and free energy of the clays.

Generally, the ion exchange and swelling process would occur simultaneously; however, capturing this complex process using current computational methods is a challenge. Hence the process is divided into two separate steps; (1) ion exchange in presence of surrounding reservoir and (2) swelling assuming that ion-exchange is completed. Similar assumption has been used by several researchers and they probed ion exchange and swelling separately. For example, Rotenberg and coworkers^10^ has studied ion exchange free energy between interlayer zone and surrounding in montmorillonite (MMT) for a fixed interlayer spacing containing two water layers. They found that there is no significant barrier (activation energy) for the exchange of cations (from outside to inside of the interlayer zone), however, insertion of anion in the interlayer zone is not favourable. After the cation exchange, the clay nanopore would re-adjust to equilibrium interlayer spacing by the exchange of water molecules with the reservoir, as recently shown in the experimental study^11^. The stability of clays, their rheological properties, and, mobility of confined water and ions are significantly affected after clay swelling^12^. Hence knowledge of clay swelling is important for its efficient utilisation in varied applications.

Early work of Hofmann et al.^13^ had established the swelling of MMT due to water adsorption in the interlayer spaces using X-ray diffraction (XRD) technique. Since then several studies have probed swelling of clays in presence of water vapors (at various relative humidities, RH), liquid water reservoir and electrolyte solution^11,14–36^. Reviewing all of them is a challenge, hence, we will focus on few studies that probed swelling behaviour with different cations. Mooney et al.^17^ studied adsorption of water vapor at various RH in cation exchange MMT (M-MMT) and found that the amount of water adsorbed at RH = $50\%$ is highest in H-MMT ($\sim 200$ mg of water/g of clay), followed by Li- and Na-MMT ($145-135$ mg/g) and smaller quanitities in K, Rb, and Cs-MMTs ($105-95$ mg/g). Berend et al.^18^ also studied water adsorption in MMT with several exposed cations and found one water layer (1WL) at lower RH and two water layers (2WLs) at higher RH in Li-, Na-, and K-MMT. The swelling behaviour of K-, Rb-, and Cs-MMT is similar and shows narrow hysteresis loop, whereas for Li- and Na-MMT, the change in basal spacing behaviour shows multiple jumps, highlighting the role of hydration energy of cations^18^. Similar observation of 1WL and 2WLs in Li-, Na-, and K-MMT has been observed in recent water vapor adsorption studies as well^19,20^. Norrish^14^ studied MMT swelling due to various ion and found both crystalline (interlayer spacing less than 20 Å) and osmotic swelling (continuous swelling beyond 20 Å) in presence of reservoir of both, pure water and electrolyte solution of corresponding cations. He found that monovalent cations of higher hydration energy exhibits higher swelling whereas monovalent ions of lower hydration energy and divalent ions with significantly higher hydration energy shows lesser swelling. He claimed that structural arrangement and interaction of confined water with oxygens of silica sheet of clay plays an important role in swelling of clays. He proposed that the free energy of the clay swelling should be a balance of (a) energy required to separate the clay sheets and (b) hydrtaion of ions; however, no explicit calculations were performed. Posner and Quirk^21^ also studied clay swelling in presence of electrolyte solutions of several mono and divalent cations using XRD. They also observed change in interlayer spacing from 2WLs to 3WLs for Li- and Na-MMT. Further, a collapse of clays to lower interlayer spacing was observed with increase in concentration of electrolyte solution. Using the law of mass action, they calculated the free energy change for swelling corresponding to 1WL change (i.e., nWLs $\rightarrow$ (n+1)WLs) and found to be directly related with the hydration energy of cation. In a recent study, the confined fluids were visualised using transmission electron microscopy and found that K$^+$ ions remain close to the surface of MMT, whereas Na$^+$ ions are located in the center of the interstitial zone also leading to more number of confined WLs^11^.

In the atomic force microscopy (AFM) and surface force apparatus (SFA) experiments, the forces exerted on the clay surfaces in presence of electrolyte solution is directly measured. At larger interlayer distances, the force profile is very well explained by the DLVO theory^34,37,38^, whereas at smaller distances, the repulsive region is attributed to hydration forces. Several groups have performed AFM and SFA experiments of many cations at different electrolyte concentrations^30,31,35,36,39–45^ and the general observations are that at low concentration snap-in (joining of two surfaces) is observed, whereas repulsive hydration force is observed beyond a critical concentration of electrolyte. For example, Pashley^34^ observed the repulsive hydration forces beyond $4\times 10^{-5}, 10^{-3}, 10^{-2},$ and $6\times 10^{-2} M$ electrolyte concentration of K$^+$, Cs$^+$, Na$^+$, and Li$^+$ cations, claimed to be associated with the size, hydration energy and surface concentration of adsorbed cations on clay surface. At higher electrolyte concentrations, oscillatory force profile with significant minimas and periodicity of 2.5 Å  is observed which was claimed to be due to dehydration of cations^35,39^. In a recent AFM study^40^, higher repulsive hydration strength and oscillation in force profile for Li$^+$ and Na$^+$ cations as compared to K$^+$ and Rb$^+$ cations were observed. Further, the mechanical energy required to bring the AFM tip closer to the surface was found to be directly correlated with the hydration energy of the cations^40^.

Computational studies of clay swelling have generally used either Monte Carlo (MC) or molecular dynamics (MD) technique to probe equilibrium water loading, interstitial spacing, and structure and dynamics of confined water and ions^16,22,46–64^. Here, we discuss those studies where different cations were probed to elucidate their role in clay swelling. Most of these simulations are performed at different water loading to obtain the equilibrium interlayer spacing and clay-hydration energies (energy change due to addition of water in dry clay) to comment on the swelling capacity of studied clays. For example, Boek et al.^48^ observed a significantly higher attractive clay-hydration energy for Na- and Li-MMTs whereas a repulsive behaviour was observed for K-MMT. Due to different hydration energies of the cations, they were found to be located either closer to the surface (K$^+$ ions) or in the center of the pore (Li$^+$ and Na$^+$ ions). Recently Teich-McGoldrick et al.^55^ performed serveral MD simulations and found that the clay-hydration energy exhibited a broad minima at 1WL for Na- and Cs-beidellite (BDL = MMT with framework charge located at silica tetrahedral sheet) whereas a steeper minima at 1WL is observed for Mg- and Ca-BDLs. At smaller interlayer spacing corresponding to 1WL, the Na$^+$ and Cs$^+$ ions are located closer to the BDL surface and at higher basal spacing of 2WLs, an additional layer of the Na$^+$ ions at the center is observed, whereas Cs$^+$ ions are located closer to the BDL surface. Most of the studies have commented on swelling capacity of clays based on the clay-hydration energy, however, only few studies have calculated the complete thermodynamics of the system. Whitley and Smith^58^ performed MC simulations to obtain thermodynamics of swelling behaviour in Na-, Cs-, and Sr-MMT. They found that the grand potential (also referred here as swelling free energy) exhibits multiple minima with global minimum located at 1WL for Cs-MMT and 2WLs for Na- and Sr-MMT indicating that hydration energy and valency has significant effect. They further comment that pressure in the confined system was correlated with the hydration number of confined ions. Recently, Shen and Bourg^63^ have performed MD simulations to obtain free energy between two MMT colloidal particles at various NaCl salt concentration in presence of explicit water. In pure water case, they found the free energy minima at three locations with global minima located at 6 Å. The above studies reproduces the experimentally observed minima in free energy profile corresponding to 1, 2, and 3WLs as well. At higher salt concentrations, they do not observe the free energy barrier between crystalline and osmotic swelling. While these studies investigated the structure of confined water and ions, clay-hydration energy in detail; however the individual pair interactions i.e., ion-surface, water-surface, and surface-surface interactions have not been investigated in details. Our recent study^65^ and that of Shen and Bourg^63^, and Li et al.^64^ have probed these individual interactions and found a significant role in the swelling free energy of clays. In our recent work^65^, we probed the swelling behaviour of mica clay where interstitial cations (K$^+$, Rb$^+$, and Cs$^+$ ions) were having similar hydration properties. Our analysis found that even 0.1 Å  difference in size of these cations leads to significant changes in swelling pressure, free energy and hence percentage of swellings.

**Table 1:** Hydration properties of Na$^+$, Li$^+$, and H$^+$ ions in bulk-liquid water.

Ions	$\Delta G_{hyd}$	$r_{max}$	$r_{min}$	$C_{n}$
Na$^+$	$-$ 369.86	2.38	3.25	5.95
Li$^+$	$-$ 474.04	2.0	2.80	4.49
H$^+$	$-$ 1050.0	2.0	2.50	2.00

$\Delta G_{hyd}$ (kJ/mol), hydration energy of ion in bulk-water from Ref.^66,67^; $r_{max}$ and $r_{min}$(Å), location of the first maximum and minimum of ion-water g(r); $C_{n}$, coordination number^68,69^.

Our earlier study motivated us to investigate the effect of significant changes in cation properties on thermodynamics of clay swelling. We have chosen muscovite mica as the reference clay and considered three cations—Na$^+$, Li$^+$, and H$^+$ ions—as the interstitial ions. These ions were chosen because they have significant difference in hydration energy and coordination number due to differences in their sizes (see Table 1). We have performed grand canonical MC (GCMC) simulations of water adsorption from the implicit bulk-liquid reservoir at various interlayer spacing (pore width, *d*). From the simulation configurations, we calculated disjoining pressure exerted on the mica framework—total as well as individual contribution of confined water, cations and opposite mica framework—from which swelling free energy (total and individual contribution) was evaluated. We found significant differences in swelling free energy which was strongly related to the structure and hydration status of confined ions. We explained these observations from the analaysis of ion-hydration (due to surface and water), sharing of hydration shells of ions by water molecules, density profiles of water and ions, and orientational analysis of water molecules. In the next section we describe the simulation methodology in brief, followed by results and discussions and finally we provide the summary and few conclusions.

**Figure 1:**
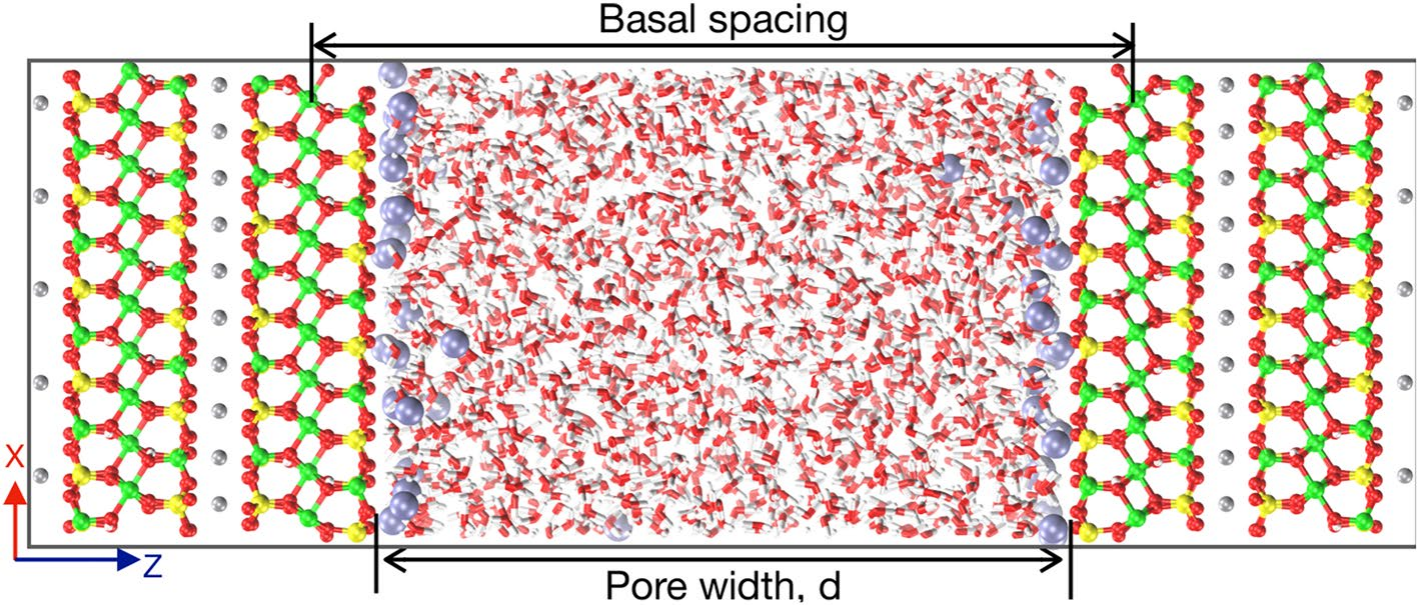
Simulation cell of mica pore containing two mica surfaces (on left and right) confining water molecules, and interstitial cations. The interlayer zone of width *d* is in equilibrium with bulk-reservoir of water (implicitly) which allows for exchange of water molecules between pore and reservoir. Color legend: Gray—potassium (K), red—Oxygen (O), green—aluminum (Al), yellow—silicon (Si), cyan—exchanged interstitial cation (i.e., either sodium, lithium or hydrogen ion) and water—licorice.

## Simulation details

We have used the previously developed computational procedure^52,65^; hence, here we describe it in brief, and details are provided in supplementary information (SI). We created mica surface of dimesnions $52.048 \times 54.108 \times 18.13$ $\hbox {\AA}^3$ with naturally occuring $\hbox {K}^+$-ions from the X-ray data^70^. The exposed $\hbox {K}^+$ ions of the top surface was replaced by M$^+$ (=Na$^+$, Li$^+$, or H$^+$) ion to create M-mica surface, whereas rest of the atoms (referred here after as framework) was kept unchanged. The confined simulation cell (Fig. 1) was created by keeping the two surfaces opposite to each other, such that framework atoms are in registery. We performed GCMC simulations to obtain equilibrium water loading in confined mica pore in the range of $d=4-40$ Å. All the simulations were started with an empty pore and a minimum of $10^9$ equilibration and $10^9$ production moves were performed at temperature of T = 298 K. Five MC moves—(1) displacement of ion and (2) insertion, (3) deletion, (4) rotation and (5) displacement of water molecule—were performed with equal probability. The mica framework atoms, water molecule, Na$^+$ and Li$^+$ ion, and H$^+$ ion were modelled using CLAYFF^71^, SPC/E^72^, Joung and Cheatham III^66^, and CHARMM^73^ forcefields, respectively. These forcefields have reproduce experimental data on swelling of clays^71,74,75^, adsorption of water on clay^68,76^; thus confirming their validity for current simulation. The thermodynamics and structure of confined systems were analysed by calculating disjoinning pressure ($\Pi$, pressure exerted on mica framework minus bulk pressure of water reservoir), swelling free energy ($\Delta \Omega ^{ex} (d)$) , one-dimensional density distribution ($\rho _z$), orientational distribution $(P(\cos \theta ,z))$, pair correlation function (g(r)) and hydration number (of ion with water: $C_{n,\hbox {W}}$, and oxygen of mica surface: $C_{n,\hbox {OS}}$) details of which are given in SI. We calculated the total $\Delta \Omega ^{ex}$ as well as individual contribution from water ($\Delta \Omega ^{ex}_{\hbox{W}}$), ions ($\Delta \Omega ^{ex}_{\hbox{I}}$) and mica framework of other side ($\Delta \Omega ^{ex}_{\hbox{S}}$). All the swelling free energy ($\Delta \Omega ^{ex}$) were normalized by thermal energy ($k_BT$) and area of the mica framework ($A_{xy}$), thus it has units of $\hbox {nm}^{-2}$ and its numerical value represent the energy required to separate clay-minerals of unit area (compared to the thermal energy). The hydrogen-hydrogen (HH) and dipole vector orientational distribution of water molecules ($P(\cos \theta _{HH},z)$ and $P(\cos \theta _{D},z)$) were calculated by considering the left side mica surface (in Fig. 1) as the reference surface, thus these distributions are respectively symmetric and anti-symmetric (i.e., $P(\cos \theta _{HH},z) = P(-\cos \theta _{HH},z)$ and $P(\cos \theta _{D},z) = P(-\cos \theta _{D},-z)$).

Since cation hydration by confined water and oxygen of mica surface place a crucial role in thermodynamics of swelling, we explain the calculation of ion-hydration in detail here. We evaluated pair correlation function between ion and oxygen of water or oxygen of mica surface ($\hbox {g}_{ {\hbox {I-W/OS}}}$(r)). The total number of water molecules or oxygen of mica surface within the first minima ($r_{min}$) of the $\hbox {g}_{ \hbox {I-W/OS}}(r)$ was evaluated as coordination/hydration number, $C_n(d)$. This hydration structure of ion was analyzed for all cations at each pore width and the $C_n$ values for $d_{\infty }$ system (i.e., mica pore system with very large pore-width having bulk-like characteristics) was considered as the reference value. Instead of reporting the absolute values of $C_n(d)$, we report the normalized values as $\hbox {f}_i = C_{n,i}(d)/C_{n, \hbox {W+OS}}(d_{\infty }) \times 100$, which provides the information on change in hydration structure due to confinement. The subscript $i=$ W, OS and W+OS in $\hbox {f}_i$ and $C_{n,i}$ represent the normalised and absolute hydration number of cation due to water, surface oxygen, and total, respectively. During hydration analysis (discussed later) we found sharing of hydration shell where some of the water molecules hydrating a cation also simultaneously hydrates nearby cations. We identified and calculated number of such water molecules which are hydrating only one ($C_{n, \hbox {1W}}$) and simultaneously two ($C_{n, \hbox {2W}}$) cations and reported in normalized form as $\hbox {f}^{i  \hbox {W}} = C_{n,i  \hbox {W}}(d)/ C_{n,  \hbox {W}} (d_{\infty }) \times 100$. In case of a water molecule hydrating two cations, they could be from the same side of mica framework or opposite sides; percentages of which ($\hbox {f}^{ \hbox {2W}}_{ \hbox {S}}$ and $\hbox {f}^{ \hbox {2W}}_{ \hbox {O}}$) were also calculated same as $\hbox {f}^{ \hbox {2W}}$. For the $d_{\infty }$ system, the two surfaces are apart and hence $\hbox {f}^{ \hbox {2W}}_{ \hbox {O}} (d_{\infty }) = 0$. We also found (discussed later) that the cation were hydrated by only one, simultaneously two or three oxygens of mica surface and for their easy identification, we refer to them as $\hbox {M}_{i \hbox {OS}}$ and their percentages are reported as $\chi ^i$ ($i=1,2,$ or 3). To get a three dimensional (3D) picture of the hydration shell of these cations, we calculated the three-dimensional g(r) within the $r_{min}$ (details given in SI) and represented as $\hbox {g}_{ \hbox {I-W/OS}}(x,y,z)$.

**Fig﻿ure 2:**
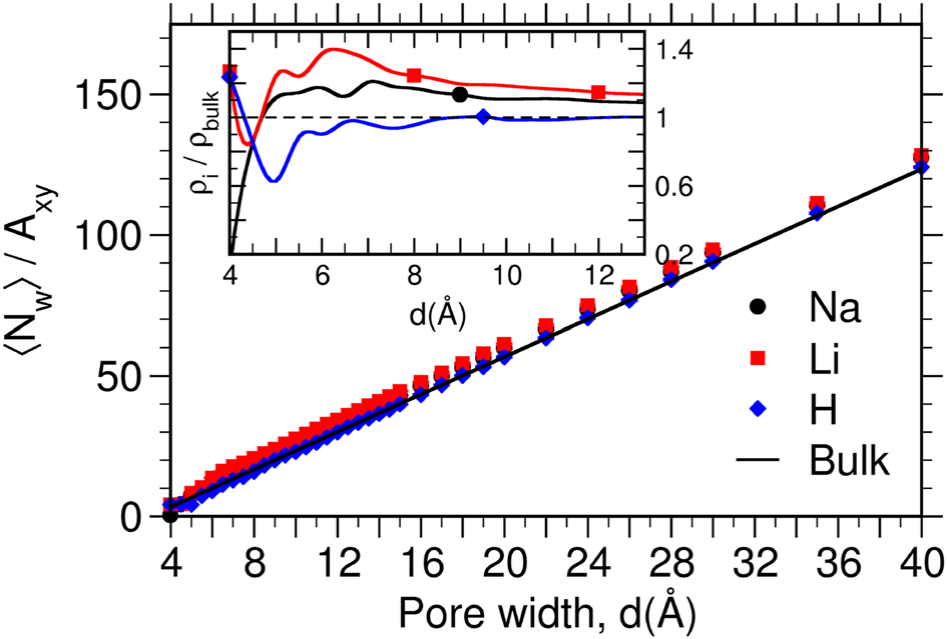
Number of water molecules adsorbed ($N_W$) per unit area of the mica surface ($A_{xy}$) in (black circle) Na-, (red square) Li-, and (blue diamond) H-mica pore at various pore widths (*d*). The solid line is the number based on bulk density of water (i.e., $\rho _b = 0.0334$ $\hbox {\AA}^{-3}$). Inset shows the significant deviations in density of confined water ($\rho _i$ normalized by $\rho _b$) at smaller *d*.

## Results and discussion

### Equilibrium water loading

Figure 2 shows the equilibrium water loading ($N_W$) in M−mica pores obtained from GCMC simulations at various *d*. The water content in all mica systems shows a qualitatively similar trend, however, they are quantitatively different. We observe water molecules of $N_W \sim 11$, 120 and 120 in Na-, Li-, and H-mica system even at $d = 4$ Å, which is the interstitial width of anhydrous (dry) naturally occurring K-mica. This indicates that mica clay with these ions is hygroscopic in nature which adsorbs water molecules even under extreme confinement. The water loading increases continuously for larger pores of $d > 4$ Å. We observe that the number of adsorbed water molecules are significantly higher in Na- and Li-mica system than bulk for corresponding *d* (i.e., data points are above the linear line of bulk system in in Fig. 2). This difference is better represented in density of confined water ($\rho _i=N_W/V_{av}$, where $V_{av} = V_{p} - V_{excl}$, is the available pore volume, $V_p = A_{xy}d$, is the pore volume, $V_{excl} = A_{xy}\zeta$, is the excluded volume due to rigid wall, and $\zeta$ is the thickness of the excluded zone near mica surface obtained from study of water adjacent to single mica surface shown in Fig. S3 of SI). The inset of Fig. 2 shows significant deviation in $\rho _i/\rho _b$ form unity at smaller *d* in the range of $d = 4.5 - 12$ Å  and it asymptotically approaches unity beyond $d=12$ Å. This clearly signifies that effect of mica confinement is strongly experienced at smaller *d*. Since H$^+$ ions have smaller size and higher hydration energy compared to Li$^+$ and Na$^+$ ions (Table 1), we expected that more water molecule would adsorb in the H-mica pores compared to Na- and Li-mica pores. However, we observed a counter-intuitive behavior that $\rho _i$ follows the trend of $\rho _{ \hbox {Li}} (d)>$ $\rho _{ \hbox{Na}} (d)>$ $\rho _b>$ $\rho _{ \hbox {H}}(d)$.

**Fig﻿ure 3:**
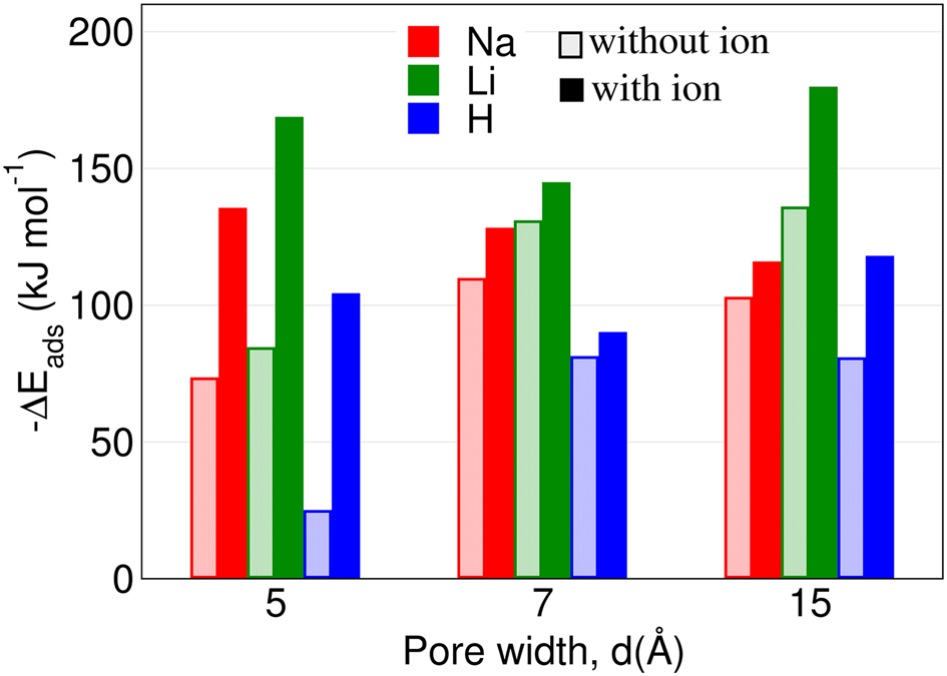
The adsorption energy of a water molecule in representative pore widths of M-mica systems.

To understand the reason, we investigated the adsorption energy of water molecules in these confined pores. The prominent adsorption sites of mica clay are (1) the ditrigonal cavity (hexagonal type of cavity formed by Si–O–Si bonds) with surface cation, (2) ditrigonal cavity without cation, and (3) regions where water co-hydrate multiple ions simultaneously. Among these adsorption sites, we have calculated the adsorption energy of single water molecules at former two adsorption sites (i.e., mica cavity with and without cations) for representative pore widths. Those water molecules which hydrate more than one ion would have significantly higher adsorption energy as compared to single ion case. Hence we did not study those cases and investigated only the limiting cases. The details of adsorption energy calculation is given in Section ﻿S2.5 in SI and the concise data obtained for various ions is shown in Fig. 3. We observe that the adsorption energy $(-\Delta \hbox {E}_{ads})$ is higher in mica cavity with ions for all the M-mica studied. Further the trend of $-\Delta \hbox {E}_{ads}$ is similar to the trend of $\rho _i$, suggesting that water adsorption in mica pore is favoured by enthalpic interactions.

**Figure 4:**
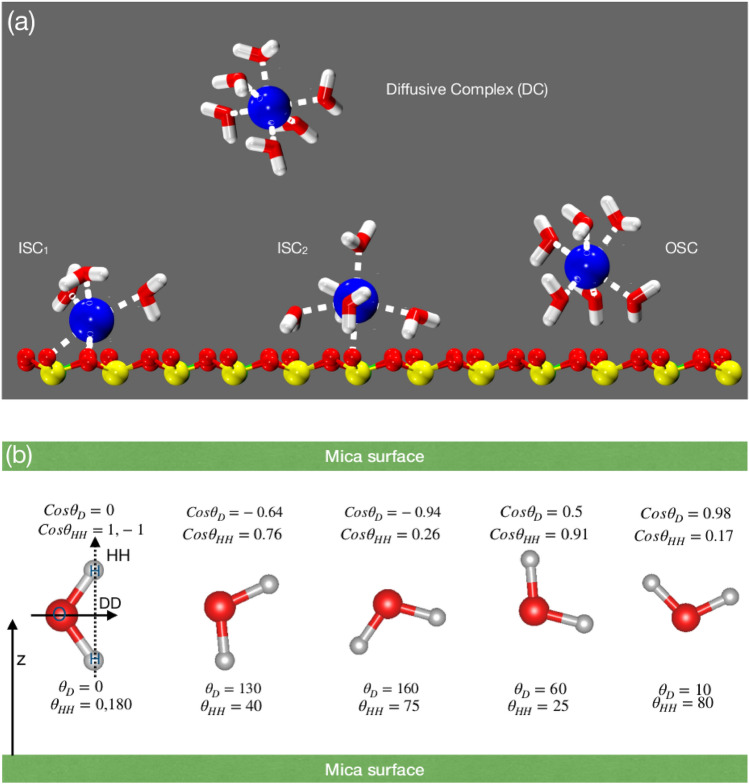
(**a**) Schematics illustrating the various ion hydration complexes observed. Inner sphere complex (ISC) where cation forms hydration shell with both surface oxygen and water molecules. $\hbox {ISC}_1$ is closer to the surface as compare to $\hbox {ISC}_2$. Outer sphere complex (OSC) where cation is located one water diameter away from surface and interact with the surface via a hydration water molecule. Diffuse ion complexes which does not interact with surface neither directly nor via hydration water. (**b**) Representative configuration of water molecules observed in confined mica pore having various orientations. $\theta _D$ and $\theta _{HH}$ are the angles formed by surface normal ($e_z$) with dipole and HH vectors, respectively.

### Structure of ions and water molecules

In literature^77–81^, including our previous work^69,82^, it has been found that cations tend to adsorb in multiple hydration states at the mineral water interface. Based on surface and water hydration illustrated in Fig. 4a, these are classified as (1) inner-sphere complexes (ISCs): where ions directly hydrate the surface oxygen and water molecules, (2) outer-sphere complexes (OSCs): where cation is located around one water diameter away from surface and interact via hydration water with the surface, and (3) diffusive complex (DC): where the cation do not interact with the surface, neither directly nor through hydration water. The layering in interfacial water (i.e., location and peak intensity in water density profile) is significantly affected by the type of cations. For the easy identification, WLs are referred to as L1, L2 and so on (smaller numeral indicates WL closer to the surface). Due to confinement, the hydration state of ions and water layering could alter significantly, especially at smaller *d* due to overlap of interfacial zones and variation in water loading (Fig. 2). We will explain the water and ion structure in mica pore from smaller to larger pore width in Na-mica system first and then discuss Li- and H-mica system.

**Figure 5:**
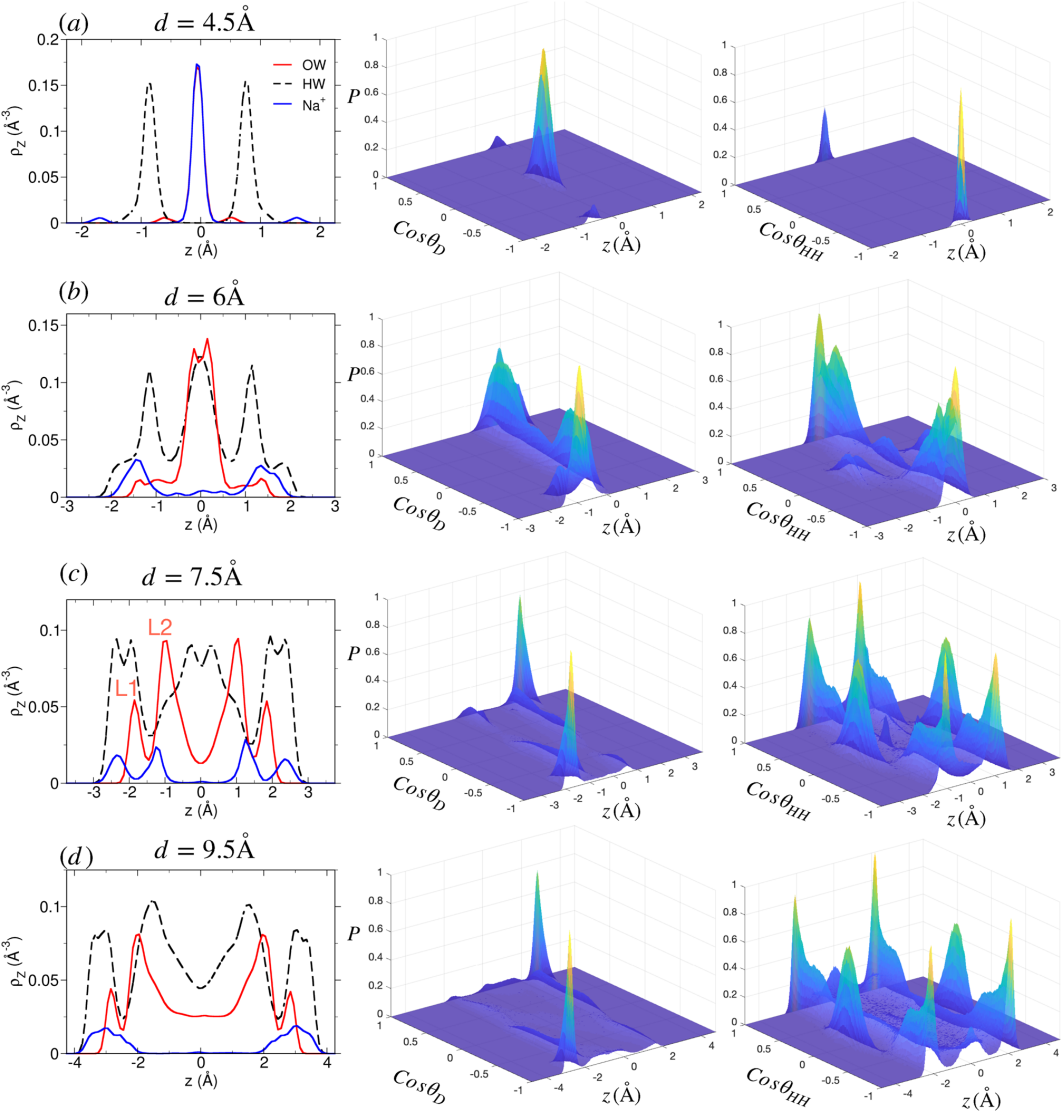
Structure of adsorbed water and cation in the Na-mica pores. (Left column) Density profiles of oxygen (red solid line) and hydrogen (black dashed line) of water molecules, and cations (blue solid line) for various pore widths of Na-mica system. In $d=4.5$ Å, the oxygen (water) density profile is overlapped by the Na$^+$ ion density. Note the redistribution of Na$^+$ ions and layering of water with increase in *d*. L1 and L2 are the water layers (WLs) located closer to the surface. Probability distribution of $\cos \theta _D$ (middle column) and $\cos \theta _{HH}$ (right column) at various *z*-position within the pore. The $P(\cos \theta _i,z)$ reported were normalized by the maximum value observed in that confined system.

#### Structure of ions and water in Na-Mica system

We found that Na$^+$ ion and water density in confined Na-mica pore is significantly different at select pore widths of $d= 4, 6, 7.5$ and 9.5 Å  and the pore width in-between these represent the transition zones, which is distinctively captured by our equilibrium thermodynamic simulations. The mica pores of transitions zones are not explained in detail here; however data is provided as Fig. S4 of SI. At $d=4.5$ Å, the cations and water molecules, both, are located at the center of the pore forming a single layer (Fig. 5a). We observe that water molecules are oriented such that oxygen is in the center and two hydrogens are pointing towards two mica surfaces where dipole vector and HH vector of water molecule is parallel and perpendicular to the surfaces making $\cos \theta _{D} = 0$ and $\cos \theta _{HH} = \pm 1$, respectively (see water configurations in Fig. 4b). Between $d = 4$ to 6 Å, the Na$^+$ ions migrate towards the surface and at $d=6$ Å, we observe a cation layer adjacent to each surface with smaller fractions of Na$^+$ ions in the center of pore (Fig. 5b). These ions form ISCs with confined water which forms a central layer; however we observe a split in the water density peak indicating a beginning of formation of separate WLs at higher pore widths. The orientation of water molecules at $d=6$ Å  exhibits a mix distribution. An intense peak at around $\cos \theta _{D} = 0.5-1$ is observed which corresponds to those water molecules that are adsorbed between mica surface and Na$^+$ ions such that they are hydrating Na$^+$ ions and water hydrogens are forming hydrogen bond with the surface oxygen aotms, resulting in $\cos \theta _{HH} = 0.5-1$ (Fig. 4b). In addition to those, few water molecules hydrate more than one Na$^+$ ions simultaneously which gives rise to $\cos \theta _{D} = \cos \theta _{HH} \approx 0$ (Fig. 5b). At $d= 7.5$ Å, the Na$^+$ ion density near surface splits into two peaks, albeit, both forming ISCs with water molecules ($\hbox {ISC}_1$ is closer to surface than $\hbox {ISC}_2$). Similarly, the water molecules also exhibits two individual peaks, L1 and L2, adjacent to each mica surface (Fig. 5c). The L1 water molecules are those which are adsorbed in ion-free cavities of mica surface with water hydrogen forming hydrogen bonds with surface oxygen atoms due to which they make $\cos \theta _{D} = \pm 1$ and $\cos \theta _{HH} = 0$ (Fig. 4b). The $P({\cos \theta _D})$ of L2 water molecules is much broader and distributed, however, the $P({\cos \theta _{HH}})$ exhibits strong peaks in the range of $\cos \theta _{HH} = 0.25-1$ (Fig. 5c). The reason for the above observation is the formation of hydrogen bonds between water molecules of two L2 layer. At $d = 9.5$ Å, we observe the merging of the $\hbox {ISC}_1$ and $\hbox {ISC}_2$ peaks of Na$^+$ ions forming a broader peak having a 2 Å  width of the distribution (Fig. 5d), where ions are found to be in multiple hydration states with surface oxygens (i.e., wider distributions of Na$^+$ ions having 1–6 surface oxygen atoms in its hydration shell), as observed in water adjacent to single mica surface shown in Fig. 14 of Adapa et al.^69^ In between $d=7.5 - 9.5$ Å, a gradual change in ion density is observed where merging of $\hbox {ISC}_1$ and $\hbox {ISC}_2$ peaks with formation of ion peak at the center of pore forming OSCs for $d=8-9$ Å  pores is also observed (see Fig. S4 of SI). We observe no significant change in the L1 and L2 layer for $d=7.5-9.5$ Å; however the width of central bulk-like region develops during these pore widths. Beyond $d = 9.5$ Å, no significant variation in the ion and water density is observed, except that a weaker layer of water molecules (L3) and bulk-like zone (L4) is observed (see Fig. S4 of SI). Water molecules of L3 layer neither hydrate the ions nor interact directly with mica surface and acts as a buffer layer between structured L2 and bulk-like L4 layer. Due to its unique position, water molecules of the L3 layer have mostly bulk-like behavior albeit a weaker structuring due to formation of hydrogen bonds with water of L2 layer.

**Figure 6:**
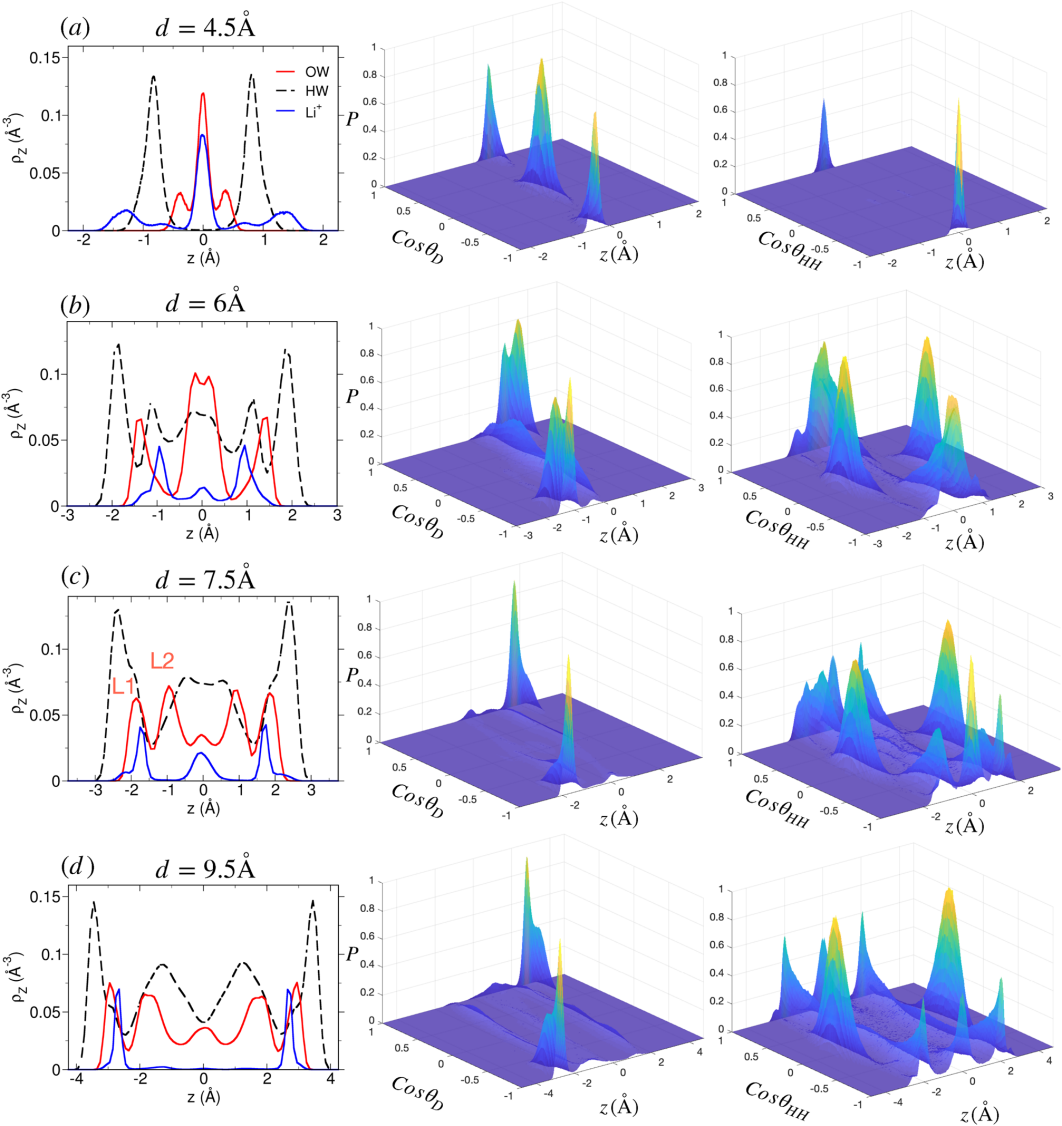
Structure of confined water and Li$^+$ ion at various *d* in Li-mica system. Legends are same as explained in Fig. 5. Note the difference in density of Li$^+$ ions and Na$^+$ ions of Fig. 5.

#### Structure of ions and water in Li-mica system

The density distribution of water and cation in Li-mica pore (Fig. 6) is qualitatively similar to that of Na-mica system (Fig. 5). We only report the cases where significant variation from Na-mica system is observed. At $d=4.5$ Å, we observe that Li$^+$ ions are adsorbed mostly at the center of the pore, however, a smaller fraction of ions are adsorbed closer to the surface as well (Fig. 6a). Similarly, three layers of (oxygen) water molecules with majority of them present in the center is also observed. The Li$^+$ ions located in the center are hydrated by water molecules whose oxygen atoms are also located at the center of pore with two hydrogen atoms pointing towards the two mica surfaces, respectively, giving rise to $\cos \theta _D = 0$ and $\cos \theta _{HH} = \pm 1$ (see Fig. 4b). The smaller fraction of Li$^+$ ions located near the surface have hydrating water located opposite to the ions with oxygen pointing towards the cation and hydrogen pointing towards the oxygen of mica surface giving rise to $\cos \theta _D = \pm 1$. At $d=6$ Å, we observe three WLs—two L1 layer near each surface and one L2 layer in the middle—and similarly three ion layers—two layers near the surface forming ISCs and one layer in the middle of the pore forming OSCs (Fig. 6b). A significant fraction of water molecules in L1 layer are located closer to the surface compared to Li$^+$ ions and also hydrate them which is absent in the Na-mica system. The water molecules of L1 layer are adsorbed in both type of mica cavities (i.e., with and without cations) with orientation as explained above (i.e., $\cos \theta _{D} \approx \pm 1$ and $\cos \theta _{HH} \approx 0$). The orientation of water in L2 layer is similar to that observed in Na-mica at similar pore widths. For $d=6-7.5$ Å  pores, the water density develops from three (L1-L2-L1) to five (L1-L2-L3-L2-L1) layer configuration (with a small shoulder of L3 layer) due to increased water loading (Fig. 6c) as similarly observed in Na-mica system (Fig. 5b, c); however, no significant change in the Li$^+$ ion density is observed as found in Na$^+$ ion density at corresponding *d*. We do not observed significant change in orientation of L1 and L2 water molecules from $d=6-7.5$ Å; however, due to presence of central Li$^+$ ion layer, the $P(\cos \theta _{HH},z)$ of L3 layer exhibits a weak structuring. For $d > 7.5$ Å, the structure of Li$^+$ ion and water is similar to that of Na-mica system, except that the density distribution of Li$^+$ ion peak is intense and narrow (Fig. 6d) as compared to Na$^+$ ion.

**Figure 7:**
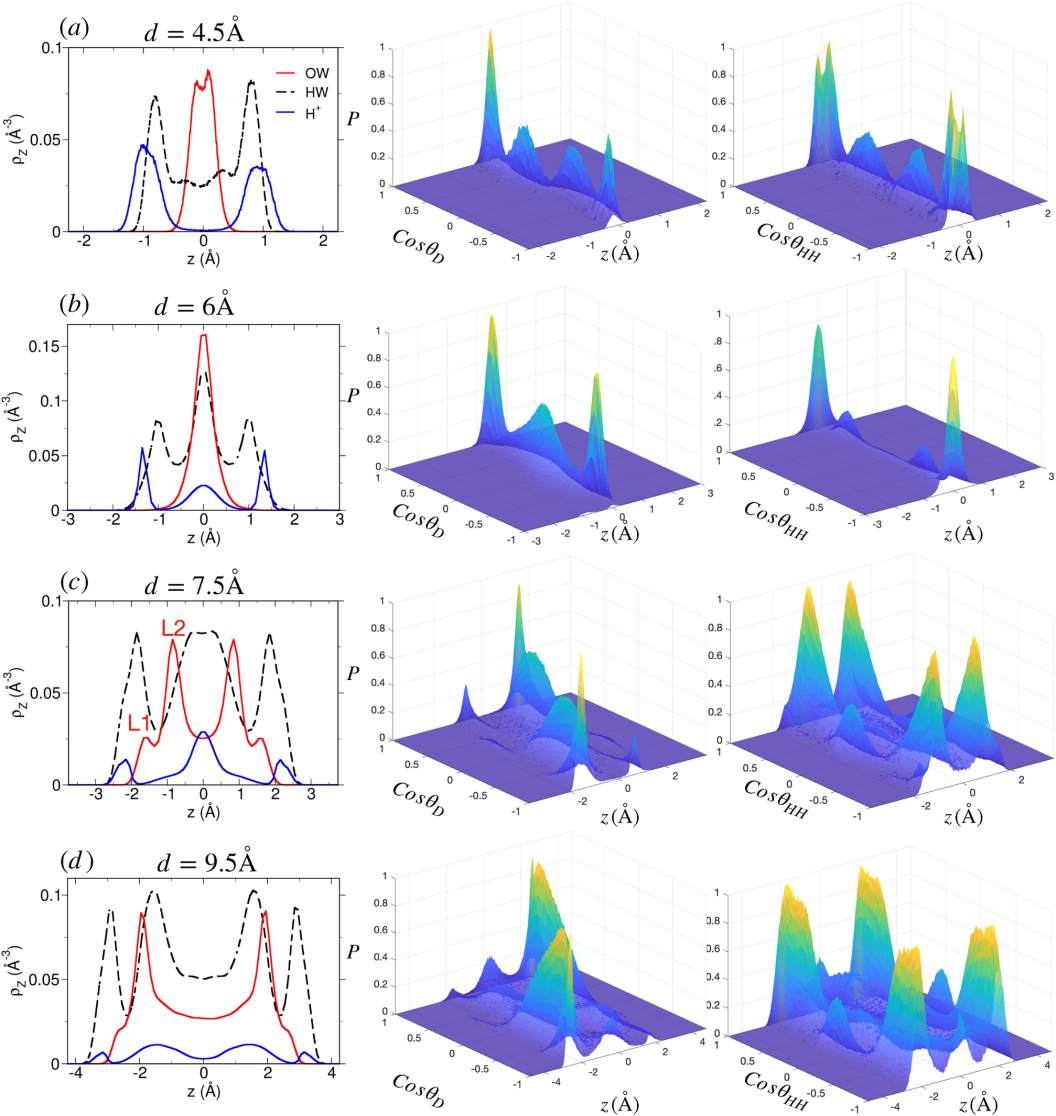
Structure of confined water and H$^+$ ion at various *d* in H-mica system. Legends are same as explained in Fig. 5. Note the difference in density of H$^+$ ions and water compared to Na- and Li-mica system shown in Figs. 5 and  6.

#### Structure of ions and water in H-mica system

Unlike in Na- and Li-mica systems, the cations in H-mica system are observed as two peaks (adjacent to surfaces) even at narrow pore width of $d = 4.5$ Å  (Fig. 7a). The H$^+$ ions are located near one of the surface oxygen atoms of the ditrigonal cavity of one side of the mica framework, whereas, the hydrating water molecule is located on the cavity of other side of framework. The orientation of these water molecules is such that oxygens are pointing towards cation and hydrogens towards mica surface ($\cos \theta _D =\pm 1)$. These ions form ISCs with adsorbed water molecules. In addition to that we observe a fraction of water molecules having orientation such that $\cos \theta _D = \pm 0-0.5$ which is also due to hydration of ions, but due to smaller size of ions having more flexibility in orientation. Between $d=4-6$ Å, an additional cation density in the center of the pore develops along with increase in loading of water molecules in the center of the pore (Fig. S6 of SI and Fig. 7b). The orientation of water molecules is governed by the location of H$^+$ ion. When the water molecules hydrate the H$^+$ ions located closer to the surface, they exhibit distributions in $\cos \theta _{D}$ around $\pm 1$ and $\cos \theta _{HH} \approx 0$; whereas, those water molecules hydrating H$^+$ ions located slightly away from the surface exhibit distributions of $\cos \theta _{D} \approx 0.5$ and $\cos \theta _{HH} \approx \pm 1$ (Fig. 7b). The analysis of snapshot indicates that H$^+$ ions adsorb near the exposed oxygen atoms of mica framework and not in the center of cavity region as observed for Na$^+$ ions reported in earlier work (see Fig. 5 of Adapa et al.^69^). At $d=7.5$ Å, we observe that water molecules are adsorbed in two (L1 and L2) layers adjacent to each surface (Fig. 7c); however the peak intensity of L1 layer is significantly smaller than (i) L2 layer of H-mica, (ii) L1 layer of Na-mica, and (iii) L1 layer of Li-mica system. Further, the fraction of H$^+$ ion (which forms OSCs) is higher compared to ions located near the surface forming ISCs (Fig. 7c). The orientation of L2 water molecules is mainly due to hydration of H$^+$ ions. The density distribution of H$^+$ ions is significantly different compared to Na$^+$ and Li$^+$ ions in the corresponding mica system at similar pore widths. Between $d=7-9.5$ Å, both ion and water density profile develops which represent the transition states, where the central cation layer breaks down in two individual layer adjacent to each surface forming OSCs with adsorbed water. While the width of central bulk-like region of water increases, albeit the densities of L1 and L2 layer remain intact (Fig. S6 of SI and Fig. 7d). Beyond $d = 9.5$ Å, the development of WLs are similar to Na-mica system, however we observe significant fractions of H$^+$ ions in diffuse state (Fig. 4) with uniform density distribution in the central zone of the mica pore. Due to hydration of H$^+$ ions forming OSCs and DCs in these mica pores, we observe weak structuring exhibited in $P(\cos \theta _{HH},z)$ in the central region as well.

**Figure 8:**
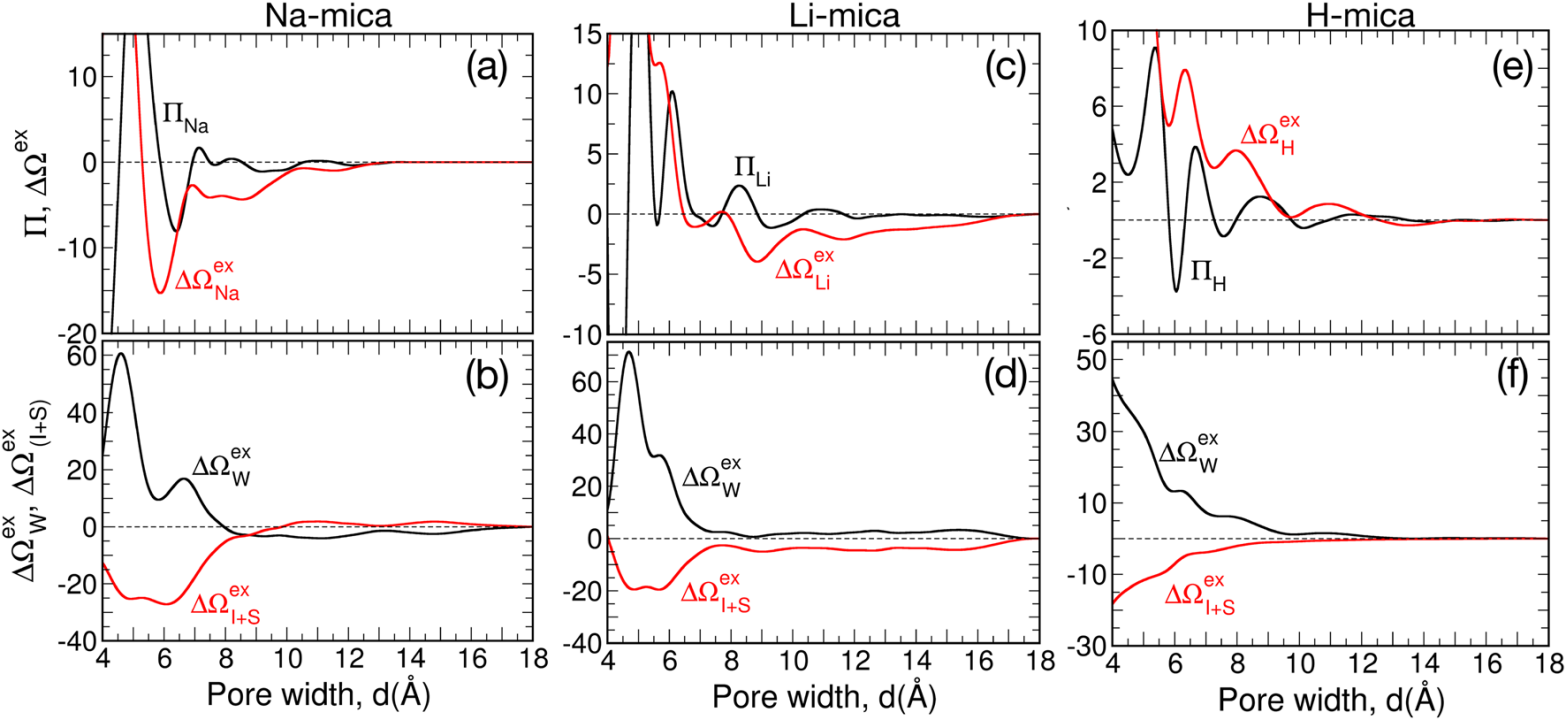
Thermodynamics of clay swelling due to water adsorption in (**a**, **b**) Na-mica, (**c**, **d**) Li-mica and (**e**, **f**) H-mica pores. (Top row) Profiles of disjoining pressure ($\Pi$, in kbar) and swelling free energy ($\Delta \Omega ^{ex}$, in $\hbox {nm}^{-2}$) in M-mica systems. With increase in hydration energy of cations from Na$^+$ to H$^+$ ion, the $\Delta \Omega ^{ex}$ progressively becomes replusive thus favouring swelling. (Bottom row) Individual contribution to $\Delta \Omega ^{ex}$ from confined water ($\Delta \Omega^{ex}_{ \hbox{W}}$) and cation + mica framework ($\Delta \Omega ^{ex}_{ \hbox{I+S}}$). The long tail in $\Delta \Omega ^{ex}$ for $d \ge 11$ Å  arises due to fluctuations in $\Pi$ which can be assumed to be zero (i.e., $\Pi (d \ge 11 {\hbox {\AA}}) = 0$). All values of $\Delta \Omega ^{ex}$ (individual contribution and total) were normalized by thermal energy ($k_BT$) and $A_{xy}$ of mica surface used, thus has units of $\hbox {nm}^{-2}$. The numerical value represent the energy required to separate clays of unit area (of $\hbox {nm}^2$) compared to thermal energy.

### Thermodynamics of water adsorption in M-mica pores

Figure 8 shows the obtained disjoining pressure ($\Pi$) and swelling free energy ($\Delta \Omega ^{ex}$) profile for Na-, Li- and H-mica system. In previous studies of simple fluids confined between planar surfaces^83–89^ the periodic oscillation in $\Pi (d)$ and hence $\Delta \Omega ^{ex}$ arises due to sequential change in number of fluid layers. The maxima in $\Pi (d)$ corresponds to compact and dense arrangement of ‘*n*’ fluid layers and subsequent minima in $\Pi (d)$ with both increase or decrease in *d* leads to formation of diffuse layers of fluids inside pores. Similar behaviour is observed here also, however due to hydrogen bonding capacity of water and hydration of ions these oscillations are not observed at integer multiple of size of the water molecules. The pore widths at which $\Pi = 0$ are the configurations where the mica framework are in equilibrium with the confined fluid (reported in Table S2 of SI). For example, these configurations in Na-mica system are found to be at $d = 5.9 (\approx 6), 6.95 (\approx 7), 7.5, 7.91 (\approx 8), 8.5, 11.5,$ and $\ge 13.5$ Å. At larger pore widths, the two confining surfaces are separated by a pool of bulk-fluid and interfacial effect of surface do not affect, hence $\Pi = 0$. Further, the fluctuations in $\Pi$ for $d > 11$ Å  is minimal; hence can be assumed to be zero. Among these configurations (where Π = 0), the pore widths which occur at the (i) repulsive branch of $\Pi$ (i.e., ${\mathbf{d}}\Pi /{\mathbf{d}}d < 0$) and (ii) attractive branch of $\Pi$ are the stable and unstable configurations, respectively. These are better illustrated in $\Delta \Omega ^{ex}$ profile, where an optima in $\Delta \Omega ^{ex}$ (minima for repulsive branch and maxima for attractive branch) is observed for each of these *d*, as $\Pi = -(\partial \Delta \Omega ^{ex}/\partial d)_{\mu ,T,A}$. We find that the overall profile of the $\Pi (d)$ and hence $\Delta \Omega ^{ex}(d)$, as well as, location of global minimum $(d_{min})$ in $\Delta \Omega ^{ex}$ is different in Na-, Li- and H-mica system indicating that hydration energy and size of interstitial ions have significant effect on swelling of clay. Further we also observe that the magnitude of $\Delta \Omega ^{ex}$ profile shifts upwards (i.e., repulsive interaction between surface increases) with increase in attractive hydration energy of ion in the order of Na$^+$, Li$^+$, and H$^+$ ion. Since the stability of such clay and swelling behaviour is dictated by the global minima in $\Delta \Omega ^{ex}$, hence we probe confined system at $d_{min}$ first, in the order of Na-, Li-, and H-mica system, then we discuss the $\Delta \Omega ^{ex}$ profile.

**Table 2:** Hydration statistics of cations and swelling free energies at $d_{min}$ and $d_{\infty }$ for M-mica system.

Property	Na-mica		Li-mica		H-mica	
	$d_{min}$	$d_{\infty }$	$d_{min}$	$d_{\infty }$	$d_{min}$	$d_{\infty }$
*d* (Å)	5.9$\approx$6	26	8.85$\approx$9	26	15	26
$C_{n, \hbox {W+OS}}$	6.33	6.11	4.20	4.18	2.02	2.02
$C_{n,{ \hbox {W}}}$	3.30	3.44	3.18	3.18	1.92	1.95
$C_{n,{ \hbox {OS}}}$	3.03	2.67	1.02	1.01	0.1	0.07
$\hbox {f}^{ \hbox {1W}}$	50.45	81.48	99	99.65	96.11	98.9
$\hbox {f}^{ \hbox {2W}}_{ \hbox {S}}$	14.36	19.92	1	0.24	2.37	1.4
$\hbox {f}^{ \hbox {2W}}_{ \hbox {O}}$	32.46	0	0	0	0	0
$\chi ^0$	0	4	7	5	90	93
$\chi ^1$	7	10	85	89	10	7
$\chi ^2$	12	24	8	5	0	0
$\chi ^{\ge 3}$	81	62	0	0	0	0
$\Delta \Omega ^{ex}$ ($\hbox {nm}^{-2}$)	$-$15.30	0	$-$3.97	0	$-$0.03	0
$\Delta \Omega ^{ex}_{ \hbox{W}}$ ($\hbox {nm}^{-2}$)	9.66	0	0.73	0	0.18	0
$$\hbox {f}^{ \hbox {2W}}= \hbox {f}^{ \hbox {2W}}_{ \hbox {S}} + \hbox {f}^{ \hbox {2W}}_{ \hbox {O}}; \Delta \Omega ^{ex} = \Delta \Omega^{ex}_{ \hbox{W}} + \Delta \Omega ^{ex}_{ \hbox{I+S}}$$						

### Confined mica systems at $d_{min}$

#### Na-mica system

**Figure 9:**
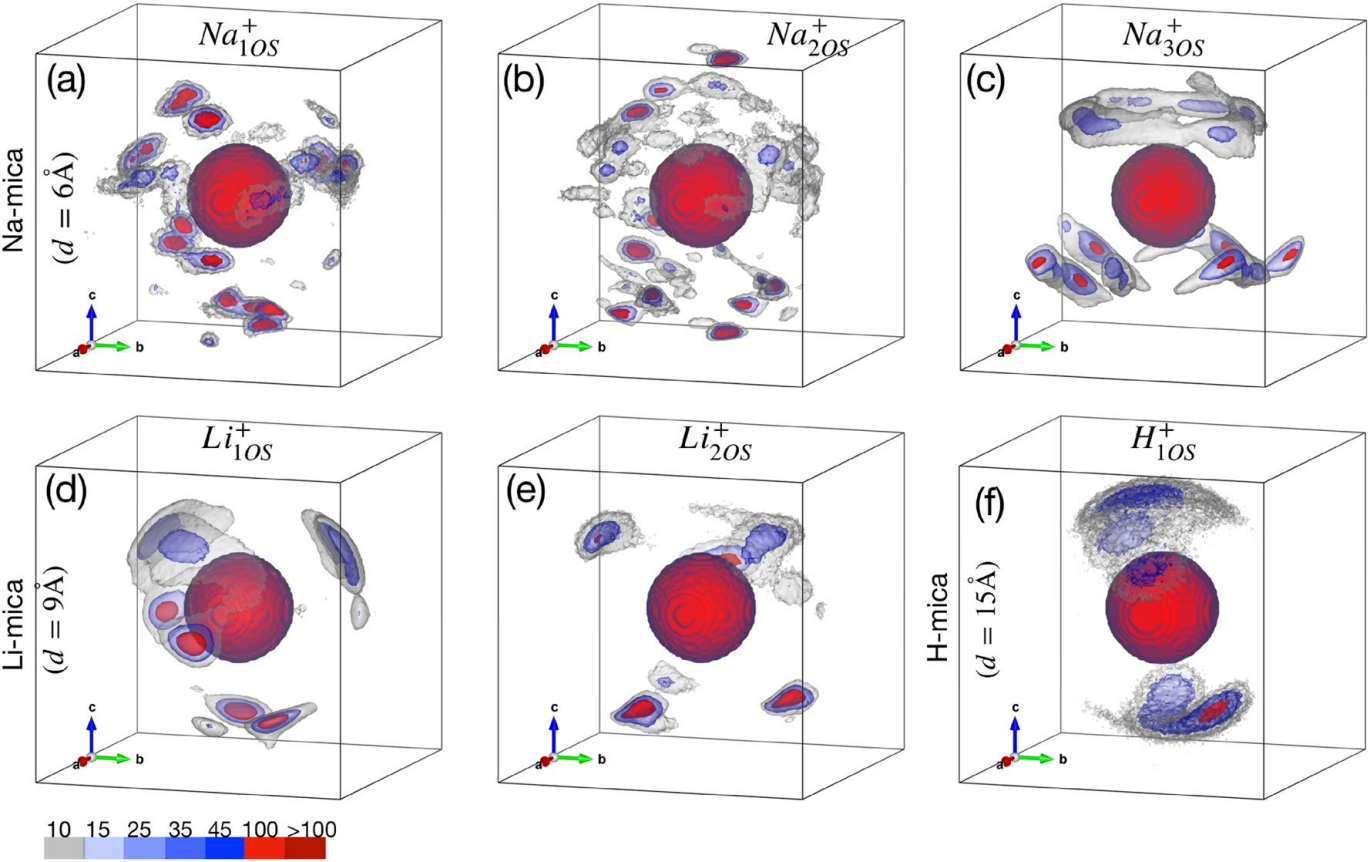
Hydration structure of cations in M-mica systems. Three-dimensional ion-oxygen (of water + mica) pair correlation function, $\hbox {g}_{ \hbox {I-W/OS}}(x,y,z)$, evaluated within the $r_{min}$ for cations hydrated by only one (M$^+_{ \hbox {1OS}}$ ), simultaneously two (M$^+_{ \hbox {2OS}}$ ) and three or more (M$^+_{ \hbox {3OS}}$ ) surface oxygens. The local density in $\hbox {g}_{ \hbox {I-W/OS}}(x,y,z)$ was normalised by $\rho _b$ for easy comparison across all cases. Iso-surfaces for selected values are shown by different semi-transparent colours (legend at bottom left). The central (red) sphere represent cation and density contour above and below the equator are due to oxygens of water and mica, respectively. When cation hydration by surface atom is more and stronger (as in $\hbox {Na}_{ \hbox {3OS}}^+$), the hydration by water is mobile and distributed forming corona above the cation. Conversely, when hydration water is strongly adsorbed near $\hbox {Na}_{ \hbox {1OS}}^+$ and $\hbox {Na}_{ \hbox {2OS}}^+$ ions, the hydration by surface atom is lesser and diffuse. In $\hbox {Li}_{ \hbox {1OS}}^+$ ion case, the peaks just below the equator is due to water. The distributed peak below $\hbox {H}_{ \hbox {1OS}}^+$ ion is due to mobility of cation.

**Figure 10:**
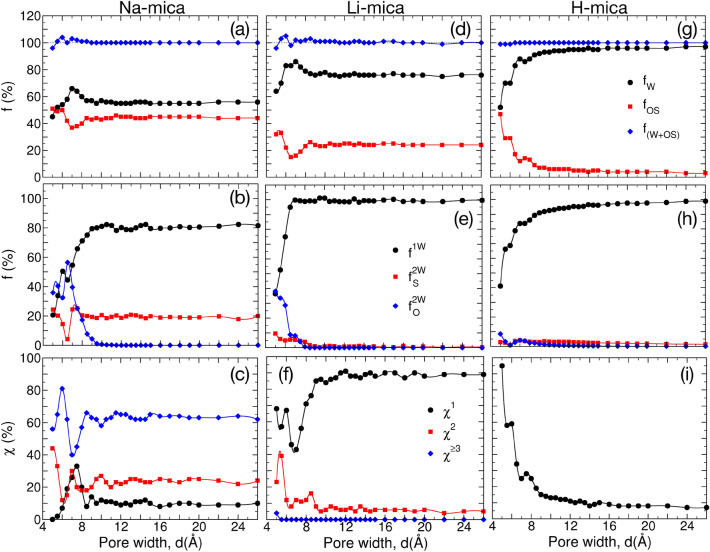
Detailed analysis of (top row) cation hydration shell, (middle row) water sharing in cation-hydration, and (bottom row) cation-hydration by surface atoms for (**a**–**c**) Na$^+$, (**d**–**f**) Li$^+$, and (**g**–**i**) H$^+$ ions in respective M-mica system. (Top row) Variation of normalized hydration number due to water ($\hbox {f}_{ \hbox {W}}$—black circle), oxygen of surface ($\hbox {f}_{ \hbox {OS}}$— red square), and total ($\hbox {f}_{ \hbox {W+OS}}$— blue diamond) with respect to *d*. (Middle row) Percentage of water molecules hydrating only one cation (i.e., no sharing, $\hbox {f}^{ \hbox {1W}}$—black circle), two cations of same side (i.e., lateral sharing, $\hbox {f}^{ \hbox {2W}}_{ \hbox {S}}$—red square), and two cations of opposite side (bridge formation, $\hbox {f}^{ \hbox {2W}}_{ \hbox {O}}$— blue diamond). (Bottom row) Percentage of cations hydrating only one surface oxygen ($\chi ^1$— black circle), simultaneously two surface oxygens ($\chi ^2$— red square), and three (or more) surface oxygens ($\chi ^{\ge 3}$— blue diamond). Percentage of cations not hydrating surface oxygens and are forming OSCs or DCs is $\chi ^0 = 100 - \sum _{i>0} \chi ^i$.

The global minimum is observed at $$d_{min} \approx 6$$ Å  with a very steep minima in the $$\Delta \Omega ^{ex}$$. The analysis of individual contribution of $$\Delta \Omega ^{ex}_{ \hbox{W}}$$ and $$\Delta \Omega ^{ex}_{ \hbox{I+S}}$$ (Fig. 8b) shows that this minimum is due to strong attraction between interstitial ions and oppositely charged mica framework atoms, while a repulsive free energy interaction is observed due to confined water. From our detailed analysis, we found two major reasons: (i) location of ions together with water, and, (ii) hydration structure of ion. In the structural analysis (as discussed in section “Structure of ions and water molecules” and Fig. 5), we find that Na$$^+$$ ions are located adjacent to individual surfaces at $$d=6$$ Å, while the confined water is present in the center of the pore as single layer. Further, we found that around 90% of adsorbed water molecules hydrate the Na$$^+$$ ions. To understand comprehensively, we probed in detail the hydration of Na$$^+$$ ions by surface oxygen atoms and confined water molecules (Table 2). We found that the hydration number of Na$$^+$$ ions is $$C_{n, \hbox {O+WS}} (d_{min})=6.33$$, which is 4 and 6% higher than $$C_n$$ for $$d_{\infty }$$ and bulk-water system (Table 1), respectively. Further breakup of $$C_n$$ shows that each Na$$^+$$ ion has an average of $$(C_{n, \hbox {W}} =)\, 3.30$$ water molecules and $$(C_{n, \hbox {OS}} =)\, 3.03$$ surface oxygen atoms in its hydration shell. We also calculated the percentage of Na$$^+$$ ions hydrated by one, two and three (or more) surface oxygens simultaneously −represented as $$\hbox {Na}^+_{ \hbox {1OS}}$$, $$\hbox {Na}^+_{ \hbox {2OS}}$$, and $$\hbox {Na}^+_{ \hbox {3OS}}$$ ions− and found them to be 7, 12 and 81%, respectively. We found that 25% of Na$$^+$$ ions are hydrated by 4 surface oxygens simultaneously, which are clubbed together and reported as $$\hbox{Na}^+_{ \hbox {3OS}}$$ ions. The larger size of Na$$^+$$ ions favour formation of hydration shell with more surface oxygen and hence Na$$^+$$ ions fit in the ditrigornal cavities of the mica surface. Indeed the $$\hbox{Na}^+_{ \hbox {3OS}}$$ ions are located closer to the surface viz-a-viz $$\hbox{Na}^+_{ \hbox {2OS}}$$ and Na$$^+_{ \hbox {1OS}}$$ ions. The adsorption of larger percentage of Na$$^+$$ ions near to the mica framework screens electrostatic interactions and thus reduces the the electrostatic repulsion between the framework (having similar +ve charge) and therefore increases the overall attractions leading to minimum in $$\Delta \Omega ^{ex}_{ I+S}$$ and $$\Delta \Omega ^{ex}$$. Further, the sharing analysis of hydration shell shows that $$\hbox {f}^{ \hbox {2W}} = 46.82\%$$ out of which $$\hbox {f}^{ \hbox{2W}}_{ \hbox{O}} = 32.46\%$$ which holds the mica frameworks of opposite sides together by hydration bridge. Similar water bridge by direct hydration of ions located on opposite sides of pore were observed in K-mica system in our recent work^65^, and others too^56^. We also calculated $$\hbox {g}_{ \hbox{I-W/OS}}(x,y,z)$$ of Na$$^+_{i  \hbox{OS}}$$ ions with both water oxygen and surface oxygens as shown in Figure 9. The central sphere represent cation and the density contour above the equator is due to the water adsorbed in pore region and density contour below the equator is due to oxygens of mica surface. The $$\hbox {g}_{ \hbox {I-W/OS}}(x,y,z)$$ of Na$$^+_{ \hbox {3OS}}$$ ions exhibits multiple strong peaks (i.e., peaks below the ion in Fig. 9c) due to fixed positions of surface oxygens whereas the hydrating water molecules form a broad-distributed corona (i.e., above the cation in Fig. 9c). These water molecules are mostly those which are hydrating only one Na$$^+$$ ion at a time. The broad distribution of water density around such ions indicates that the entropy of these water molecules is significantly higher than those water molecules which are adsorbed in tight zones hydrating multiple ions simultaneously. The Na$$^+_{ \hbox {3OS}}$$ ions acts as anchor and attracts the other mica surface through water molecules. It was further confirmed by the variation in percentage of Na$$^+_{ \hbox {3OS}}$$ ion around $$d_{min}$$ exhibiting a steep global maxima (Fig. 10), which is responsible for the steep global minima observed in $$\Delta \Omega ^{ex}$$. This indicates that $$\Delta \Omega ^{ex}$$ at $$d_{min}$$ is favored by the enthalpic interactions due to hydration of Na$$^+$$ ions by surface oxygens predominantly. On the contrary, the $$\hbox {g}_{ \hbox {I-W/OS}}(x,y,z)$$ of Na$$^+_{ \hbox {1OS}}$$ and Na$$^+_{ \hbox 
{2OS}}$$ ions exhibits (Fig. 9a, b) multiple but distributed peaks below the ion indicating that these Na$$^+$$ ions are not bound to the surface as strongly as Na$$^+_{ \hbox {3OS}}$$ ions. Further, the peaks of hydrating water molecules are slightly above the Na$$^+$$ ion and majority of these water molecules are hydrating more than one ions simultaneously, especially water molecules hydrating Na$$^+_{ \hbox {1OS}}$$ ions (Fig. 9a). Due to stronger enthalpic attraction with the two Na$$^+$$ ions, the adsorption position as well as the orientation of these water molecules is restricted, which leads to repulsion in the $$\Delta \Omega ^{ex}_{ \hbox{W}}$$, as observed.

#### Li-mica system

The global minimum is observed at $d_{min} \approx 9$ Å  due to a local minimum in $\Delta \Omega ^{ex}_{ \hbox{I+S}}$ while repulsive interaction is observed for $\Delta \Omega ^{ex}_{ \hbox{W}}$ (Table 2). Due to higher hydration energy and smaller size, the Li$^+$ ions form smaller and denser hydration shell with oxygen atoms. Further, the hydrating atoms (oxygen of water) occupy fixed position relative to each other to prevent repulsive overlap. This is manifested in $\hbox {g}_{ \hbox {I-W/OS}}(x,y,z)$ where narrow and dense peaks at selected locations are observed, unlike distributed regions observed in hydration shell of Na$^+$ ions (Fig. 9). The smaller size of Li$^+$ ions affect (i) the hydration by water, (ii) sharing of hydration water between two ions and (iii) hydration by surface oxygens which is manifested in hydration statistics (Table 2). We find that $C_{n, \hbox {O+WS}}(d_{min}) = 4.20$, significantly lesser than $C_n$ of Na$^+$ ion, out of which $C_{n, \hbox {W}} = 3.18$. Further, the percentage of water molecules sharing the hydration shell is (i.e., $\hbox {f}^{ \hbox {2W}} =)\, 1\%$ only; and that too with the cations of the same side (i.e., $\hbox {f}^{ \hbox {2W}}_{ \hbox {S}} = \hbox {f}^{ \hbox {2W}}$). The remaining hydrating water molecules are not shared and hydrate single ion alone (i.e., $\hbox {f}^{ \hbox {1W}} = 99\%$). Since, surface oxygen atoms are fixed in framework based on chemistry of surface, which does not coincide with the hydration location of Li$^+$ions, hence the probability of multiple surface oxygen atoms hydrating Li$^+$ ions decreases. This is manifested in the fraction of Li$^+_{ \hbox {2OS}}$ and Li$^+_{ \hbox {3OS}}$ ions which are found to be only 8 and 0%, unlike higher fractions observed in Na-mica system (Table 2). Hence, the total hydration with surface decreases to $C_{n, \hbox {OS}} = 1.02$, where 85% of Li$^+$ ions hydrate single oxygen atom. When we compare these hydration statistics with Li$^+$ hydration for $d_{\infty }$ system (Table 2), the numbers are very close, suggesting that no significant change occurs in their hydration structure for $d_{min}$ as well. These numbers are significantly smaller than observed for Na-mica systems at global minima (Table 2). In the calculated $\hbox {g}_{ \hbox {I-W/OS}}(x,y,z)$, the peaks located below the ion corresponds to surface oxygen whereas peaks just below the equator corresponds to L1 water hydrating the ions. The L2 water mostly hydrate above the ions and they also form intense and narrow 3D peaks confirming that hydrating structure is very tight. Due to limited sharing, each ion behaves as individual moiety on the surface covered by the water molecule and anchored by the surface oxygens. Further, at $d_{min}$, we observe a smaller but significant L3 layer (Fig. 6) which contains mostly non-hydrated water molecules which acts as bridging layer forming hydrogen bonded network between hydrated and non-hydrated water molecule of the system present on individual surfaces.

#### H-mica system

The $$\Delta \Omega ^{ex}$$ profile is repulsive at smaller pore widths and approaches to zero at larger pore widths due to small fluctuations in $$\Pi$$ (Fig. 8e). This suggest that formation of thick water layer between H-mica surface is favourable and stable. We considered $$d=15$$ Å  as the significantly larger pore width where the hydration structure of H$$^+$$ ion is analyzed. The size of H$$^+$$ ion is even smaller than Li$$^+$$ ion due to which its hydration number is only ($$C_{n} =)$$  2, and we found that it remains constant at all *d*. Further, we found that only 10% of H$$^+$$ ions remain close to surface having one surface oxygen in the hydration shell whereas rest of the atoms are present as diffuse ion spanning the entire width of the pore. We analyzed $$\hbox {g}_{ \hbox {I-W/OS}}(x,y,z)$$ of H$$^+_{ \hbox {1OS}}$$ ions and found one strong yet distributed peak each above and below the ions due to water and surface oxygen, respectively. Due to smaller size and lesser hydration number, these ions do not acts as anchor as like Na$$^+$$ and Li$$^+$$ ions to hold the mica surfaces together. Being attached to surface decreases their entropy while maintaining the same hydration number does not favor enthalpically either. Conversely, being in the central zone of pore with same hydration number of 2 increases its entropy, hence leads to decrease in $$\Delta \Omega ^{ex}_{ \hbox{W}}$$ with increase in *d*.

The above analysis indicates that those ions which has higher size that snuggly fits and forms larger number of hydration with surface oxygens of mineral surface acts as anchor and holds the opposite side mineral surface by forming water bridge. Such scenario was observed in recent study of water adsorption in K-, Rb- and Cs-mica pres, as well^65^. With decrease in size of ion and increase in hydration energy, the interaction with mineral surface decreases and ion hydration is favored by free water molecules, therefore such ions favor swelling in mineral surfaces. The tendency of cations to adsorb more closer to the surface with increase in cation size has been observed in recent AFM experimental studies too^40^.

### Analysis of $\Delta \Omega ^{ex}$ profile of M-mica systems

#### $\Delta \Omega ^{ex}$ of Na-mica system

We now focus on the overall $\Delta \Omega ^{ex}$ profile to understand the reason for the observed behaviour (Fig. 8a, b). To probe the system in detail, we divide the $\Delta \Omega ^{ex}$ profile of Na-mica system into subzones of $\hbox{i) } d < 6, \hbox{ii) } d=6-8.5, \hbox{iii) } d=8.5-10.5$ and $\hbox{iv) } d> 10.5$ Å. The repulsion in $\Delta \Omega ^{ex}$ for $d<6$ Å, is observed due to decrease in hydration of Na$^+$ ions as shown in Fig. 10a. Both, the number of water molecules hydrating the ions ($\hbox {f}_{ \hbox {W}}$ in Fig. 10a) as well as fractions of Na$^+_{ \hbox {3OS}}$ ions ($\chi ^{\ge 3}$ in Fig. 10c) decreases leading to decrease in enthalpic attraction resulting in repulsion in $\Delta \Omega ^{ex}_{ \hbox{W}}$ and $\Delta \Omega ^{ex}_{ \hbox{I+S}}$ (Fig. 8). The regime between $d=6 - 8.5  {\hbox {\AA}}$ corresponds to confined system between two successive minima in $\Delta \Omega ^{ex}$ with a deep minima at $d = 6$ Å, significant maxima at $d=7$ Å, and broader minima at $d=8.5$ Å. Figure 10a–c of the ion hydration shows that during this regime a significant change in cation hydration behavior by surface oxygen and water molecules occurs which is responsible for the observed behavior. The detailed analysis at $d_{min} = 6$ Å, reported in previous section, has shown that the global minimum in $\Delta \Omega ^{ex}$ occurs primarily due to maximum hydration of Na$^+$ ions with surface oxygen. The maximum in $\Delta \Omega ^{ex}$ at $d=7$ Å  occurs due to maximum hydration by water molecules ($\hbox {f}_{ \hbox {W}} = 66\%$) and minimum hydration by surface oxygens ($\hbox {f}_{ \hbox {OS}}=37\%$) especially by the significant decrease in percentage of Na$^+_{ \hbox {3OS}}$ ions (Fig. 10a, c). The structural analysis shows that between $d=6 - 7.5$ Å, water and ions change from single layer to compact double layer with rearrangement of water completing at $d=7$ Å  (Fig. 5) followed by Na$^+$ ions at $d=7.5$ Å. This transition is also reflected in $\Delta \Omega ^{ex}_{ \hbox{W}}$ which shows a local maximum at $d\approx 7$ Å  due to structuring of water, whereas continuous increase in $\Delta \Omega ^{ex}_{ \hbox{I+S}}$ is observed due to increased distance between mica surfaces and re-structuring of ions. Further, the variation in $\Delta \Omega ^{ex}_{ \hbox{W}}$ and $\Delta \Omega ^{ex}_{ \hbox{I+S}}$ is continuous between $d=7-8.5$ Å  with opposite effect (i.e., increase in attraction and repulsion, respectively) resulting in minimum at $\Delta \Omega ^{ex}$ at $d=8.5$ Å. The Na$^+$ ion hydration by water and surface oxygen is recovered during $d=7-8.5$ Å  by the adsorption of water molecules and ion-hydration shell restructuring which gives rise to $\hbox {f}_{ \hbox {OS}} = 44\%$ and $\chi ^{\ge 3} = 66\%$ (Fig. 10a, c). These analysis reconfirm above observation that Na$^+$ ions acts as anchor where multiple surface oxygen atoms hydrate the cation to hold the mica surface together via bridge formation by the confined water molecules. Further, the hydration of ions shows no significant change in individual and total behavior beyond $d = 8.5$ Å. The regime between $d=8.5-10.5$ Å, shows an increase in $\Delta \Omega ^{ex}$ with a local maximum at $d=10.5$ Å  while $\Delta \Omega ^{ex}_{ \hbox{W}}$ exhibits a broad, continuous and attractive minima. The structural analysis of confined system shows development of L3 layer from a non-singificant to fully developed layer, which acts as interfacial layer forming hydrogen bonded network across the pore widths by the adsorption of non-hydrated water molecules mainly. Beyond $d=10.5$ Å, the overall $\Delta \Omega ^{ex}$ reaches to zero asymptotically due to adsorption of bulk-like water molecules in the central zone, as evident from the change in structure of water and $\Delta \Omega ^{ex}_{ \hbox{W}}$.

#### $\Delta \Omega ^{ex}$ of Li-mica system

Similar to the Na-mica system, we also observe multiple minima in the $\Delta \Omega ^{ex}$ for the Li-mica system (Fig. 8c). The major difference is that we observe four distinct minima (at $d_1 = 5.5, d_2 = 6.85 \approx 7, d_3 = 8.85 \approx 9$ and $d_4 = 11.65$ Å) with global minimum at third location (i.e., $d_{min} = d_3$). To elucidate the reason behind these minima in $\Delta \Omega ^{ex}$, here also we divided overall profile into sub-regimes: i) $d \le 5.5$, ii) $d= 5.5-7$, iii) $d=7-9$, and iv) $d>9$ Å. The minimum at $d_1 = 5.5$ Å  in $\Delta \Omega ^{ex}$ is repulsive and occurs due to attractive contribution from $\Delta \Omega ^{ex}_{ \hbox{I+S}}$ and repulsive contribution from $\Delta \Omega ^{ex}_{ \hbox{W}}$ (Fig. 8d). The Li$^+$ ions have $C_n (d_1) = 4.29$ out of which 33% contribution arises from surface oxygen and the rest from water molecules. Further, only 5% of Li$^+$ ions are not hydrated by surface oxygen whereas 57 and 39% of ions have respectively one and two surface oxygens in their hydration shell. Among the various pore widths, the Li$^+$ ions have highest surface hydration at $d_1=5.5$ Å, which gives rise to highest attraction in $\Delta \Omega ^{ex}_{ \hbox{I+S}}$. When we compare the behaviour of $\Delta \Omega ^{ex}_{ \hbox{I+S}}$ between Na- and Li-mica system, the qualitative behaviour is similar, however, it is more attractive in Na-mica than Li-mica system due to differences in cation hydration by surface oxygens (explained further). At similar pore width, the Na$^+$ ions have $(C_{n, \hbox {OS}}=)$  3.03 surface oxygen in its hydration shell with $\chi ^{\ge 2}= 98\%$; whereas these numbers are significantly lesser -around 1.37 and 39%, respectively- in Li-mica system. The observed repulsion in $\Delta \Omega ^{ex}_{ \hbox{W}}$ (at $d_1$) is due the arrangement of water molecules in confined pore, such that oxygens form single central layer with positively charged hydrogens pointing towards the positively charged mica framework (Fig. 5). For $d < 5.5$ Å, the water adsorption and hence the hydration decreases which gives rise to repulsion in $\Delta \Omega ^{ex}_{ \hbox{W}}$ and hence $\Delta \Omega ^{ex}$, as observed (Fig. 8c, d).

The minimum in $\Delta \Omega ^{ex}$ at $d_2$ is due to local minimum in $\Delta \Omega ^{ex}_{ \hbox{W}}$ and local maximum in $\Delta \Omega ^{ex}_{ \hbox{I+S}} (d \approx d_2)$, which is completely reversal to the behvaior observed at $d_1$ (Fig. 8c, d). The main reason is the redistribution of Li$^+$ ions forming an additional central layer where Li$^+$ ions form OSCs with the water molecules (Fig. 6). Due to this redistribution, ($\chi ^0 =$) 45% of ions are not hydrated by surface oxygen and hence leads to significant dip in hydration by surface (from $\hbox {f}_{ \hbox {OS}}=33$ to $16\%$) and increase in hydration by water (from $\hbox {f}_{ \hbox {W}} = 70$ to $86\%$) while total cation hydration number is similar (Fig. 10d–f). This change in hydration is manifested in the $\Delta \Omega ^{ex}_{ \hbox{W}}$ and $\Delta \Omega ^{ex}_{ \hbox{I+S}}$. Between $d=5.5-7$ Å, adsorption of water molecules leads to transition from single WL in the center to three WLs with a transition at $d=6$ Å, where local maximum in the $\Pi (d)$ is observed due tight packing of WLs in L1-L2-L1 arrangment. In the third regime of $d = 7-8.5$ Å, we once again observe a redistribution of ions where Li$^+$ ion migrate from the center of the pore to near the mica surface along with development of L1 and L2 WLs adjacent to each surface and a common L3 WL at the center of pore. Due to migration of ions, the $\chi ^0$ decreases from 45 to 7%, which gives rise to local minima in $\Delta \Omega ^{ex}_{ \hbox{I+S}}$ at $d_3$. Further, we observed that all the additional water molecules adsorbed during this regimes do not hydrate the cations, hence the hydration of ions is not affected significantly. Only those water molecules which were hydrating more than one ions simultaneously, their number decreases due to formation of individual hydration shell around each ions. Therefore, no significant change in $\Delta \Omega ^{ex}_{ \hbox{W}}$ is observed during this regime. For $d > 9$ Å, there are still small fraction of Li$^+$ ions which are present away from the surface and with increase in pore width and adsorption of water molecules they migrate towards the surface. This leads to $\Delta \Omega ^{ex}_{ \hbox{I+S}}$ and $\Delta \Omega ^{ex}_{ \hbox{W}}$ approaching to zero asymptotically with local fluctuations, which leads to local minima in $\Delta \Omega ^{ex}$, as observed.

#### $\Delta \Omega ^{ex}$ of H-mica system

The $\Delta \Omega ^{ex}$ profile is repulsive at smaller pore widths and approaches to zero at larger pore widths due to small fluctuations in $\Pi (d)$. The individual contribution of $\Delta \Omega ^{ex}_{ \hbox{I+S}}$ and $\Delta \Omega ^{ex}_{ \hbox{W}}$ are, respectively, attractive and repulsive in nature and asymptotically approaches to zero at larger *d*. The reason for these observations could be understood from the hydration behavior of H$^+$ ions. At all *d*, we observe that $\hbox {C}_n$ of H$^+$ ions is constant with no significant increase due to hydration by surface oxygen which was seen in Na$^+$ and Li$^+$ ions. Further, due to smaller size of H$^+$ ions they hydrate only two oxygens (either of water or one of water and one of surface oxygen). By adsorbing to the surface oxygen, the H$^+$ ions do not gain an extra enthalpic contribution, while looses mobility and hence entropy. Therefore, from the free energy perspective, hydration of H$^+$ ions in bulk region of confined system is favorable. As a result, we observe that most of the ions migrate to center of the bulk and found as diffuse ions at large *d*. Therefore hydration of H$^+$ ions with surface decreases (and conversely with water increases) with increase in *d*. The reason for $\Delta \Omega ^{ex}_{ \hbox{W}}$ being repulsive is that when we decrease the pore width, we are increasing the concentration of distributed H$^+$ ions in the confined system which increases the osmotic pressure. Also, the decrease in *d* brings the H$^+$ ions closer to the surface, thus screening the electrostatic interactions and hence leading to continuous attraction between mica frameworks resulting in $\Delta \Omega ^{ex}_{ \hbox{I+S}} < 0$. The local minima in $\Delta \Omega ^{ex}$ arises due to minor fluccuations in $\Delta \Omega ^{ex}_{ \hbox{W}}$ and $\Delta \Omega ^{ex}_{ \hbox{I+S}}$ which corresponds to development of H$^+$ ions forming ISCs, OSCs and DCs with adsorbed water molecules. As mentioned earlier that oscillations in $\Pi$ (and therefore $\Delta \Omega ^{ex}$) occurs due to transition from ‘n’ ordered to - ‘n’ diffuse to - ‘n+1’ ordered layers in the confined fluid. Here also we observe similar behavior of formation of layers in ions and water. Below $d = 6$ Å, the depletion of water molecules from hydration shell of H$^+$ ions leads to repulsion in $\Delta \Omega ^{ex}_{ \hbox{W}}$ and hence $\Delta \Omega ^{ex}$. Between $d=6-7.5$ Å, we see the transition in ions and water arrangement and at $d=7.5$ Å, the H$^+$ ions are arranged such that majority of them (around 70%) are forming OSCs with two water layers formed adjacent to each mica surface. Similarly, for $d=7.5-10$ Å, we observe the transition of central cation layer forming OSCs to formation and development of two cation layer (one layer adjacent to each surface forming OSCs) along with development of interfacial water layer. At the completion of this cation layer formation, we observe the minima in $\Delta \Omega ^{ex}$ at $d \approx 10$ Å. For $d > 10$ Å, the H$^+$ ions mostly migrate to the center of pore due to favorable hydration with water molecules which gives rise to slow approach of $\Delta \Omega ^{ex}$ to zero.

## Summary and conclusion

Here we focus on understanding the role of different hydration properties of cation on thermodynamics of clay swelling by water adsorption. The clays acquire cations of different properties by cation exchange, which plays a central role in many applications such as nutrient supply in soil, oil extraction, disposal of nuclear waste, cosmetics and pharmaceuticals. The swelling capacity and stability of clay after exchange of cation of different hydration properties will change significantly and hence fundamental understanding of clay swelling is necessary for their efficient utilisation. For this we have chosen mica as the reference clay and Na$^+$, Li$^+$, and H$^+$ ions as the interstitial cations, which have significant differences in hydration properties. We performed several GCMC simulations at various pore widths ($d = 4-40$ Å) to probe water adsorption. The swelling free energy ($\Delta \Omega ^{ex}$) was calculated from the disjoining pressure ($\Pi$) −total as well as individual contribution from confined water, cations and mica framework of opposite side. We also probed structural properties of water and ions by calculating several distributions. We found that analysis of various cation hydration scenarios (e.g., by surface oxygen, water, shared water) and three dimensional pair correlation function between ion and hydrating oxygen provides valuable informations. The key findings of this works are listed as below.

- Water adsorption in Na-, Li- and H-mica pores is qualitatively similar; however significant quantitative differences are observed, specially at smaller *d*. Water molecules adsorbed even at dry conditions of mica having $d= 4$ Å, suggesting hygroscopic nature of these ions. Higher water density was expected in H-mica pores due smaller size and higher hydration energy of $\hbox {H}^+$ ions; however the counter-intuitive trend of $\rho _{ \hbox{Li}}> \rho _{ \hbox{Na}}> \rho_b > \rho _{ \hbox{H}}$ was observed at smaller *d*. The reason was adsorption energy of water molecules in these systems which followed similar trend of density indicating that mica-framework also has significant contribution in energetics of the system.
- We found multiple hydration states of confined ions and water with variation in *d*. The $\hbox {Na}^+$ ion is adsorbed in the center of the pore for $d \le 5$ forming ISCs with adsorbed water. The equilibrium adsorption position of $\hbox {Na}^+$ ion shifts towards the surfaces with increase in *d*, splitting into two layers at $d=7.5$ Å, and remerging to form broad and distributed peak adjacent to each surface at $d \ge 9.5$ Å. The Li$^+$ ions are mostly found in ISCs for all *d*, except for $d=6.5-7.5$ Å, where significant fraction of Li$^+$ ions are adsorbed in the center of the pore forming OSCs. The $\hbox {H}^+$ ions are adsorbed in multiple layer inside the pore at all *d*. Only for $d \le 6.5$ Å, H$^+$ ions are adsorbed close to surface forming ISCs whereas for the rest of the pores, majority of cations are away from the surface forming OSCs and DCs.
- In all three system, continuous evolution in water structure (preferential location and orientation) for $d \le 11$ Å  is observed due to adsorption density of water, hydration of cations, and, formation of water-water and water-surface hydrogen bonds.
- The swelling pressure ($\Pi$) and free energy ($\Delta \Omega ^{ex}$) profiles show the oscillatory behavior with diminishing to zero for $d \ge 11$ Å  in all three mica system. The location of optima in the $\Delta \Omega ^{ex}$ is different due to significant role of cations. A shift in location of global minimum of $\Delta \Omega ^{ex}$towards the higher *d* values and decrease in attractive strength of $\Delta \Omega ^{ex}$ is observed in the increasing order of hydration energy of $\hbox {Na}^+$, $\hbox {Li}^+$, and $\hbox {H}^+$ ions. In Na-mica system, the energy barrier for crystalline swelling from $d=6$ to 9 Å, is observed whereas the barrier is lower for crystalline ($d=9$ Å) to osmotic swelling ($d > 12$ Å). For Li-mica, the energy barrier for crystalline to osmotic swelling is lesser compared to Na-mica system, whereas for H-mica the $\Delta \Omega ^{ex} > 0$ for all *d* thus osmotic swelling is favoured.
- We found that cation hydration by surface atoms play a key role in deciding the swelling ability of clay. The Na$^+$ ion which has lesser hydration energy and larger size, they comfortably fit in the ditrigonal cavity of mica surface and are simultaneously hydrated by more number of surface oxygen atoms. On each side of mica frameworks, the strongly adsorbed Na$^+$ ions acts as anchors and hold the mica pore together at $d_{min}=6$ Å  by sharing of water in their hydration shell with the cations located on the other side of mica surface forming an electrostatically connected bridge of mica-Na-water-Na-mica.
- The Li$^+$ ions which has moderate hydration energy and average size are indeed hydrated by surface oxygen atoms, albeit lesser number and sharing of water in the hydration shell with nearby Li$^+$ ions is also minimum at $d_{min} = 9$ Å. Specifically the fraction of ions hydrated by multiple surface oxygen atoms simultaneously is very less at $d_{min}=9$ Å. In between $d=7-9$ Å, the cations prefer to detach from surface, adsorb in the center of the pore and maintain hydration shell with water molecules due to higher hydration energy. Only at extreme confinement of $d \le 6$ Å  the simultaneous hydration of cation by more than one surface oxygen atom is observed.
- The tendency of cation detachment from mica surface is observed in the H$^+$ ions also which has smallest size and largest hydration energy amongst the cations studied. The hydration by surface atoms and sharing of water in hydration shell, both, is minimum as the H$^+$ ions are mostly present in the center of the pore forming DCs, thus exerting a constant osmotic pressure on the mica frameworks, favouring swelling.

This detailed understanding of change in clay swelling behaviour due to differences in hydration properties of interstitial cations will provide aid in experimental analysis and design of better inhibiting materials.

## Supplementary Information

Supplementary Information.

Supplementary Information.

## Data Availability

The datasets used and/or analysed during the current study is available from the corresponding author on reasonable request.
