# Supplementary material for: Cation hydration by confined water and framework-atoms have crucial role on thermodynamics of clay swell﻿ing
Source: Sci Rep. 2022 Oct 24;12:17810. doi: 10.1038/s41598-022-21349-3 (PMC9592624; doi:10.1038/s41598-022-21349-3)
Supplement: Supplementary file 1 — Supplementary Information. [file 41598_2022_21349_MOESM1_ESM.pdf]

Supporting Information: Cation Hydration by Confined Water and Framework-Atoms Have Crucial Role On Thermodynamics of Clay Swelling

Sai Adapa[†,‡] and Ateeque Malani[a,*,†]

†*Department of Chemical Engineering, Indian Institute of Technology Bombay, Mumbai 400076, India*

‡*Research and Development Division, Tata Steel Limited, Jamshedpur, 831001, India*

E-mail: amalani@iitb.ac.in

[a]Corresponding Author: amalani@iitb.ac.in

Content

Sections:

S1	Computational Methodology	4
S1.1	Molecular Models of Mica Surface, Water, and Cations	4
S1.2	Interaction Potential Models	5
S1.3	Grand Canonical Monte Carlo (GCMC) Simulations	5
S2	Property Calculations	6
S2.1	Disjoining Pressure and Grand Potential	6
S2.2	Density Distribution	8
S2.3	Orientational Distribution	8
S2.4	Ion Hydration	9
S2.5	Adsorption Energy of Water in Mica Pores	10

Figures:

**S1:**	Simulation cell of clay-water system	4
**S2:**	Adsorption energy of water molecules in Na-, Li- and H-mica pores	10
**S3:**	Density distribution of interfacial water adjacent to mica-surface	13
**S4:**	Density distribution of water and ion at various pore widths in Na-mica system	14
**S5:**	Density distribution of water and ion at various pore widths in Li-mica system	15
**S6:**	Density distribution of water and ion at various pore widths in H-mica system	16

Tables:

**S1:**	List of symbols and abbreviations (frequently) used.	3
**S2:**	Frocefield parameters	12
**S3:**	Swelling free energies	12

References

References ??

Table S1: List of symbols and abbreviations (frequently) used.

M^+ ion	:	Na^+, Li^+ and H^+ ion
M-mica	:	Mica surface containing M^+ ions as interstitial ions
MC	:	Monte Carlo
GCMC	:	Grand Canonical Monte Carlo
MD	:	Molecular dynamics
MMT	:	Montmorillonite
WL	:	Water layer
nWL	:	'n' (number of) Water layers
A_{xy}	:	lateral area of a exposed surface
ΔG_{hyd}	:	Hydration energy of ion in bulk-water
g(r)	:	Radial distribution function (RDF)
$g_{\text{I-W/OS}}(r)$	:	RDF between ion and oxygens of water and mica surface
$g_{\text{I-W/OS}}(x,y,z)$	:	Three-dimensional RDF between ion and oxygens of water and mica surface
r_{max}	:	Location of first peak of ion-water g(r)
r_{min}	:	Location of first minimum of ion-water g(r)
C_n	:	Coordination number - number of atoms located within r_{min} of the cation
$C_{n,W}$	:	Number of water molecules located within r_{min} of the cation
$C_{n,OS}$	:	Number of oxygen atoms of mica surface within r_{min} of the cation
f^{W}	:	Fraction of water molecules in the cation hydration shell
f^{iW}	:	f^{W} that are hydrating i-ions simultaneously
f^{2W}_{S}	:	f^{W} hydrating two cations located on same side of mica framework
f^{2W}_{O}	:	f^{W} hydrating two cations located on two sides of mica framework
χ^i	:	Fraction of cations hydrated by i-surface oxygen atoms simultaneously
M^+_{iOS}	:	M cation hydrated by i-surface oxygen atoms simultaneously
d	:	Width of clay (mica) pore
d_{min}	:	Mica pore width where global minimum in $\Delta\Omega^{ex}$ is observed
d_∞	:	Mica pore with large width where confined water exhibits bulk-like behavior
$\Pi(d)$ or Π_M	:	Disjoining pressure in M-mica pore of width d
$\Delta\Omega^{ex}(d)$	:	Grand potential or swelling free energy of clay pore of width d
$\Delta\Omega^{ex}_W, \Delta\Omega^{ex}_I, \Delta\Omega^{ex}_S$	:	Swelling free energy due to confined water, interstitial ions and mica framework
$k_B T$	:	Thermal energy at temperature, T
e_z	:	Surface normal of mica surface (located on left side in Fig. S1)
e_D	:	water dipole vector
e_{HH}	:	HH vector passing through two hydrogen atoms of water
θ_D	:	Angle between e_D and e_z
θ_{HH}	:	Angle between e_{HH} and e_z
$P(\cos\theta, z)$	:	Orientational distribution of water molecules at z-location within the pore
ρ_z	:	Density of water molecule at z-location within the pore
$\rho_M(d)$	:	Density of confined water in M-mica pore of width d
ρ_b or ρ_{bulk}	:	Density of liquid (bulk) water
$N_W(d)$	:	Number of water molecules adsorbed in mica pore of width d
V_{av}	:	Available pore volume($=V_p - V_{excl}$)
V_p	:	Pore volume ($=A_{xy}d$)
V_{excl}	:	Excluded volume near mica surface ($=A_{xy}\zeta$)
ζ	:	Thickness of excluded zone near mica surface
ΔE_{ads}	:	Adsorption energy of a water molecules to be adsorbed in empty mica pore
ISC, OSC and DC	:	Inner-sphere complexes, Outer-sphere complexes, Diffusive complexes
L1, L2, L3	:	Nomenclature of water layer adjacent to mica surface

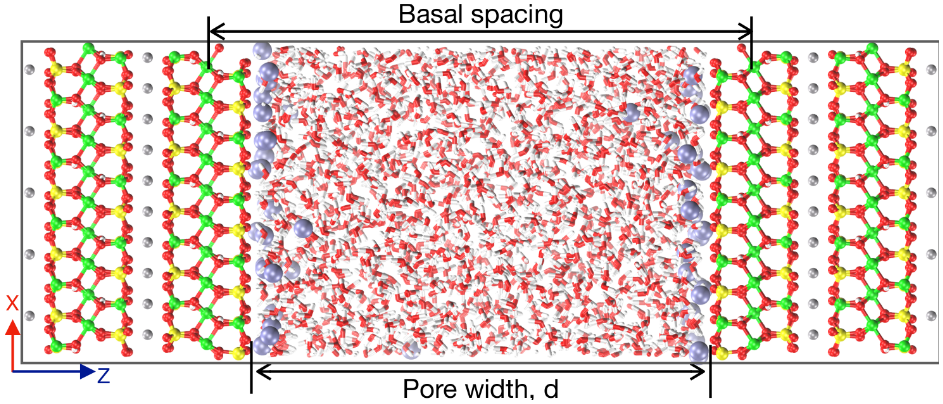

Figure S1: Simulation cell of mica pore containing two mica surfaces (on left and right) confining water molecules, and interstitial cations. The interlayer zone of width d is in equilibrium with bulk-reservoir of water (implicitly) which allows for exchange of water molecules between pore and reservoir. Color legend: Gray - potassium (K), red- Oxygen (O), green - aluminum (Al), yellow - silicon (Si), cyan - exchanged interstitial cation (i.e., either sodium, lithium or hydrogen ion) and water - licorice

S1 Computational Methodology

S1.1 Molecular Models of Mica Surface, Water, and Cations

The muscovite mica supercell containing 2 unit cells along z-direction of thickness 18.13 Å was repeated to create a mica surface of dimensions L_x = 52.048 Å and L_y = 54.108 Å ($A_{xy} = L_x L_y$ = 28.16 nm^2). The prepared surface contains two exposed surfaces (top and bottom) and one interstitial layer containing K^+ ions. The M-mica surface was created by replacing the K^+ ions of the top exposed surface to M^+ ion (M=Na^+, Li^+, or H^+). The charge on the framework is located at the exposed silica tetrahedral sheet and its magnituded is 1e per unit cell, which is different from MMT studied in literature[?,?,?] where charge is located either at octahedral, tetrahedral or both layer. The exposed cations are mobile whereas the rest of the mica surface was kept rigid for all simulations and referred hereafter as the mica framework. The mica pore was created by placing the two mica surfaces opposite to each other such that the framework atoms of both surfaces are in registry (Fig. S1). The water

molecule was modelled by the three site rigid model and primitive model for cations.

S1.2 Interaction Potential Models

The van der Waals and electrostatic interactions between two atomistic sites were modelled using Lennard-Jones (LJ) and Coloumbic interactions,[?,?,?] respectively. The Coloumbic intearaction were evaluated using three-dimensional (3D) Ewald summation with correction for slab geometry[?] as described in detail in earlier works.[?,?,?,?] The short range part of the Ewald summartion and the LJ interactions were evaluated within the spherical cut-off of 14 Å. The long-range part of the Ewald summation was evaluated in reciprocal space within the relative accuracy of 10^{-6}. A vaccum of width $d_v = \max(d + 2d_m, L_x, L_y)$ was added above each mica surface to reduce electrostatic interaction between images, making the entire width of simulation cell in z-direction to be $L_z = d + 2d_v + 2d_m$, where d is the pore width and $d_m = 18.13$ Å is the thickness of mica framework. The mica atoms, water molecule, Na^+ and Li^+ ion, and H^+ ion were modelled using CLAYFF,[?] SPC/E,[?] **?**, and CHARMM[?] forcefields, respectively. Since, the charge on H^+ ion is lesser than Na^+ and Li^+ ion, the excess charge on mica framework was managed by increasing the charges of exposed silicon and aluminium atoms of silica tetrahedral layer. The parameters are given in Table S2. These forcefields have reproduce experimental data on swelling of clays,[?,?,?] adsorption of water on clay;[?,?] thus confirming their vlaidity for current simulation.

S1.3 Grand Canonical Monte Carlo (GCMC) Simulations

The MC simulations were performed in the grand canonical ensemble (at constant μVT; μ - chemical potential of water, V - volume of the interstitial zone and T - temperature). The MC moves performed were, i) displacement, ii) rotation, iii) insertion, and iv) deletion of water molecules and v) displacement of ions with equal probability which were accepted based on Metropolis criteria.[?,?] The MC simulations were performed at various pore widths in the range of $d = 4 - 40$ Å. All the simulations were started with an empty pore and a minimum

of 10^9 equilibration and 10^9 production moves were performed at $T = 298$ K. The implicit bulk reservoir corresponds to liquid water at room temperature having density $(\rho_b =)0.0334$ Å^{-3} and the chemical potential $(\mu =) - 29.37$ kJ/mol, evaluated separately. For the faster equilibration of the simulations, we also performed MD simulations in canonical ensemble (constant NVT) intermittently during the equilibration period of GCMC simulations. The temperature of the system was maintained using Nose-Hoover thermostat and rigid geometry of the water molecules were maintained using SHAKE algorithm.[?] All mica farmework atoms were kept frozen by not solving equations of motions for them and simulations were performed for 1 ns with a timestep of 2 fs. The GCMC simulations and property calculations were performed using in-house developed codes and the MD simulations were performed using LAMMPS package.[?]

S2 Property Calculations

The thermodynamics and structure of confined water were analysed by calculating disjoining pressure (Π), swelling free energy ($\Delta\Omega^{ex}$), one-dimensional density distribution (ρ_z), orientational distribution ($P(\cos\theta, z)$), pair correlation function (g(r)) and hydration number (C_n) details of some are given below.

S2.1 Disjoining Pressure and Grand Potential

The fundamental equation which describes the thermodynamics of a confined system is given as,

$$\mathbf{d}\Omega = -P^b\mathbf{d}V - S\mathbf{d}T - N_W\mathbf{d}\mu + 2\gamma\mathbf{d}A_{xy} - (A_{xy}f_s)\mathbf{d}d\,, \tag{1}$$

where P^b is the bulk water pressure, V is the volume, S is the entropy, N_W is the number of water molecules adsorbed in the pore, γ is the fluid-wall interfacial tension, and f_s is the solvation force per unit area of the surface, A_{xy}. For the bulk water system, the last two terms are to be removed due to the absence of interface and pore. The excess grand potential

($\Omega^{ex} = \Omega - \Omega^b$) in differential form is obtained as,

$$\mathbf{d}\Omega^{ex} = -S^{ex}\mathbf{d}T - N^{ex}\mathbf{d}\mu + 2\gamma\mathbf{d}A_{xy} - (A_{xy}f_s)\mathbf{d}d, \tag{2}$$

where $S^{ex} = S - S^b$, $N_W^{ex} = N_W - N_W^b$, and superscript b indicate a property of a bulk system. At constant T, μ, and A_{xy}, we obtain

$$f_s = -\frac{1}{A_{xy}}\left(\frac{\partial\Omega^{ex}}{\partial d}\right)_{T,\mu,A_{xy}}. \tag{3}$$

In literature,[?,?,?,?,?,?,?] this net force per unit area (f_s) is referred to as the disjoining pressure, $\Pi = P^z - P^b$, where P^z is the normal component of pressure acting on the surface.

Disjoining pressure gives an insight of attraction or repulsion between two surfaces of the slit pore due to pore filling by the surrounding liquid. The negative value of force indicates that surfaces are attracted to each other, and the system wants to collapse by squeezing out the confined fluid. Conversely, the positive value of force indicates that the confining fluid pushes the surfaces; hence, squeezing out of it is not favorable but difficult. The confined system would be stable at those pore width where it is in equilibrium with the surrounding fluid (i.e., $\Pi = 0$). In our simulation, we calculated the disjoining pressure by explicitly calculating the force exerted on the mica framework due to confined water, mobile surface ions, and atoms of opposite side mica framework as,

$$F_1 = \sum_{i=1}^{N_{surf1}}\left[\sum_{j=1}^{N_W} f_{ij} + \sum_{j=1}^{N_{ion}} f_{ij} + \sum_{j=1}^{N_{surf2}} f_{ij}\right], \tag{4}$$

where f_{ij} is the interatomic force between atom i and j, N_{surf1} and N_{surf2} are the number of (fixed) atoms in the mica frameworks of left side and right side in Fig. S1, and N_{ion} are the number of free ions in the confined region. The absolute pressure exerted on the surface is $P^z = (F_1 + F_2)(z\text{-component})/(2A_{xy})$, whereas the disjoining pressure is defined as $\Pi(d) = P^z(d) - P^z(d_\infty)$, where d_∞ is the pore system with very large pore-width having

bulk-like characteristics.

To obtain the information about the stability of surface separation (at a given d) and thermodynamics of swelling, we calculate excess grand potential from Eq. 3 using Π as $\Delta\Omega^{ex}(d) = \Omega^{ex}(d) - \Omega^{ex}(d_\infty) = A_{xy}\int_d^{d_\infty} \Pi \, \mathbf{d}d$. We calculated the total Π as well as individual contribution from water (Π_W), ions (Π_I) and mica framework of other side (Π_S) from which total and individual contribution to swelling free energy were also evaluated (i.e., $\Delta\Omega^{ex}$, $\Delta\Omega^{ex}_W$, $\Delta\Omega^{ex}_I$, and $\Delta\Omega^{ex}_S$). All the swelling free energy ($\Delta\Omega$) reported in this manuscript were normalized by thermal energy (k_BT) and area of the mica framework (A_{xy}), thus the $\Delta\Omega$ has units of nm^{-2}. The numerical value of $\Delta\Omega$ represent the energy required (compared to the thermal energy) to separate clay-minerals of unit area (of nm^2).

S2.2 Denstity Distribution

The one dimensional density distirbution of confined water and ions within the pore were evlauated as $\rho_z = 1/(N_j A_{xy}) \sum_i^{N_j} \delta(z - z_i)$ where N_j is the total number of atoms (hydrogen, oxygen, or cations), z_j is the z-coordinates of selected atom, and delta function was evaluated numerically as combination of two discrete heavyside step functions ($\Theta(x) = 0$ for $x < 0$ and $= 1$ for $x > 0$) as $\delta(x) = (1/dx)(\Theta(x - dx/2) - \Theta(x + dx/2))$ where dx is the bin thickness.

S2.3 Orientational Distribution

We used dipole (e_D) and HH vector (e_{HH}) of a water molecule to define the orientation (angle θ_D and θ_{HH}) with respect to the surface normal (e_z of left side of mica surface in Fig. S1) as $\cos\theta_i = e_i \cdot e_z$. We calculated the joint probability distribution of water orientation at various location within the pore as $P(\cos\theta_i, z) = \sum_j^{N_j} \delta(\cos\theta_i - \cos\theta_j)\delta(z - z_j)$ which combines the effect of local density of water molecules in the pore. We arbitrarily defined one of the hydrogen atoms as the reference atoms, hence $P(\cos\theta_{HH}, z)$ is symmetric with respect to the $\cos\theta_{HH} = 0$. While plotting, the $P(\cos\theta_i, z)$ were normalised by the maximum value observed for given pore width.

S2.4 Ion Hydration

To understand the hydration of cations, we evaluated pair correlation function between ion and oxygen of water or oxygen of mica surface as $\mathrm{g}_{\text{I-W/OS}}(r) = 1/(4\pi r^2 N_I \rho_{\text{W/OS}}) \sum_i^{N_I} \sum_j^{N_W/N_{OS}} \delta(r - r_{ij})$, where r_{ij} is the distance between ion and oxygen atom, $\rho_{\text{W/OS}}$ is the density of water or oxygen of mica surface, N_I, N_W and N_{OS} are the number of cations, water molecules and oxygen of mica surface, respectively. The total number of water molecules or oxygen of mica surface within the first minima (r_{min}) of the $\mathrm{g}_{\text{I-W/OS}}(r)$ was evaluated as coordination/hydration number as $C_n = \int_0^{r_{min}} \rho_{\text{W/OS}} 4\pi r^2 \mathrm{g}_{\text{I-W/OS}}(r) \mathbf{d}r$. This hydration strucutre of ion was analysed for all cations at each pore width and the C_n values for d_∞ system was considered as the reference value. Instead of reporting the absolute values of $C_n(d)$, we report the normalized values as $\mathrm{f}_i = C_{n,i}(d)/C_{n,\text{W+OS}}(d_\infty) \times 100$, which provides the information on change in hydration structure due to confinement. The subscript i = W, OS and W+OS in f_i and $C_{n,i}$ represent the normalised and absolute hydration number of cation due to water, surface oxygen, and total, respectively. During hydration analysis (discussed later) we found sharing of hydration shell where some of the water molecules hydrating a cation also simultaneously hydrates nearby cations. We identified and calculated number of such water molecules which are hydrating only one ($C_{n,1\text{W}}$) and simultaneously two ($C_{n,2\text{W}}$) cations and reported in normalized form as $\mathrm{f}^{i\text{W}} = C_{n,i\text{W}}(d)/C_{n,\text{W}}(d_\infty) \times 100$. In case of a water molecule hydrating two cations, they could be from the same side of mica framework or opposite sides; percentages of which ($\mathrm{f}_{\text{S}}^{2\text{W}}$ and $\mathrm{f}_{\text{O}}^{2\text{W}}$) were also calculated same as $\mathrm{f}^{2\text{W}}$. For the d_∞ system, the two surfaces are apart and hence $\mathrm{f}_{\text{O}}^{2\text{W}}(d_\infty) = 0$. We also found (discussed later) that the cation were hydrating only one, simultaneously two or three surface oxygens and for their easy identification, we refer to them as $\mathrm{M}_{i\text{OS}}$ and their percentages are reported as χ^i ($i = 1, 2,$ or 3). To get a three dimensional (3D) picture of the hydration shell of these cations, we calculated the 3D g(r) within the r_{min} as, $\mathrm{g}_{\text{I-W/OS}}(x, y, z) = 1/(N_I \rho_b) \sum_i^{N_I} \sum_j^{N_W/N_{OS}} \Theta(r_{min} - r_{ij}) \delta(x - x_{ij}) \delta(y - y_{ij}) \delta(z - z_{ij})$ normalised by the ρ_b for easy comparison across cations.

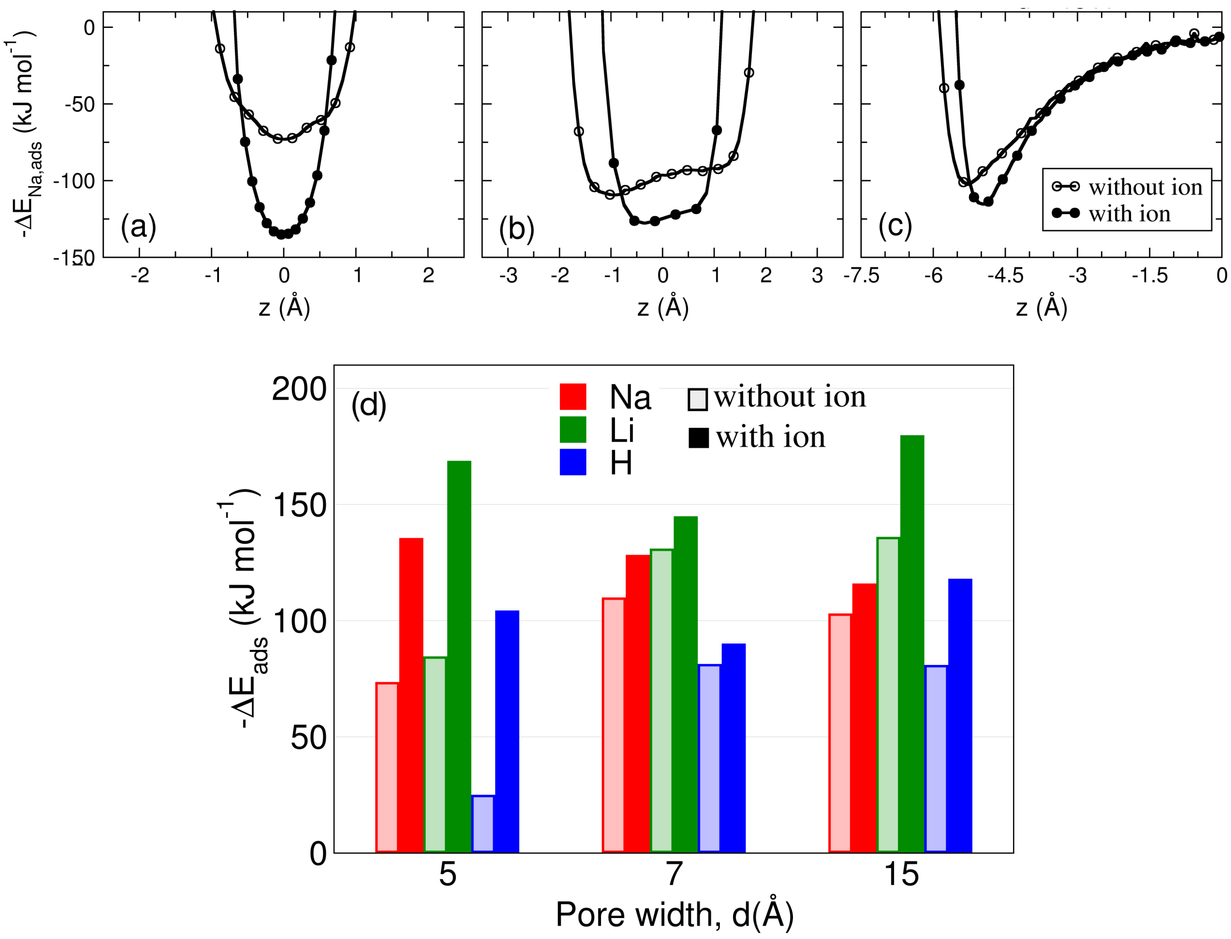

Figure S2: (a-c) Energy of single water molecule calculated at various z-position within the Na-mica pore of widths $d = 5, 7$, and 15 Å. The position of probe molecule was selected near the hexagonal cavity such that it is hydrating a cation (with ion) and not-hydrating a cation (without ion), and then interaction energy profile was calculated (see text for details). d) The minima of the water energy profile, referred as adsorption energy (-ΔE_{ads}), in representative pore widths of M-mica systems.

S2.5 Adsorption Energy of Water in Mica Pores

We investigated the adsorption energy of water molecules in these confined pores. The prominent adsorption sites of mica clay are i) the ditrigonal cavity (hexagonal cavity formed by Si-O-Si bonds) with surface cation, ii) ditrigonal cavity without cation, and iii) regions where water co-hydrate multiple ions simultaneously. Among these adsorption sites, we have calculated the adsorption energy of single water molecules at former two adsorption sites (i.e., mica cavity with and without cations) for representative pore widths. We probed

a region of $5 \times 5 \times 2$ Å^3 near a ditrigonal cavity with a grid size of 0.1 Å. At each location, the center of mass (oxygen atom) position of water molecule was fixed and 100 MC moves of rotation of water molecule were performed to obtain the minimum energy configuration within this region (x_m, y_m, z_m). Then we fixed x, y position of water (mainly oxygen) to x_m, y_m and varied the z-position with a step size of 0.1 Å. At each z-position, 2×10^4 MC moves of water rotation were performed and the last 10^4 moves were used to obtain average configurational energy of a water molecule. The energy profiles for representative systems studied is shown in Fig. S2a-c. The ion configuration was taken to be the last configuration of GCMC simulations where position of ions are non-uniform due to mobility and adsorption of water molecules. As a result, we do not observe symmetric energy profile at lower d where overlap of interaction due to nearest neighbour is higher. The minimum of this energy profile was considered as the adsorption energy $(-\Delta \mathrm{E}_{ads})$ and the collective data obtained for various ions is shown in Fig. S2d.

Table S2: Forcefield parameters of cations, mica and water atoms.

molecule	atom	ϵ, kJ mol^{-1}	σ, Å	q, e
Water†	O	0.6502	3.166	-0.8476
	H	0.0	0.0	0.4238
Mica‡	O (exposed)	0.6502	3.166	-1.05
	O (buried)	0.6502	3.166	-1.16875
	O (of OH)	0.6502	3.166	-0.95
	H (of OH)	0.0	0.0	0.425
	K	0.4184	3.334	1.0
	Si	7.7007×10^{-6}	3.302	2.1
	Al$^{\perp}$	7.7007×10^{-6}	3.302	1.575
	Al	5.5639×10^{-6}	4.2714	1.575
	Si-T*	7.007×10^{-6}	3.302	+2.2475
	Al-T*	7.007×10^{-6}	3.302	+1.7225
Surface Ions$^{\parallel}$	Na^{+}	1.47545	2.16	+1
	Li^{+}	1.409	1.409	+1
	H^{+}	0.192464	0.40	+0.41

†− SPCE Model,? ‡− CLAYFF Potential?
⊥ − Substituted aluminum in silica tetrahedral layer,
∗− exposed atoms of silica tetrahedral layer in H-mica
‖ − H - CHARMM potential? and Na, Li - **?**

Table S3: Pore widths (d in Å) at which optimum in swelling free energy ($\Delta\Omega^{ex}$ in nm^{-2}) and $\Pi = 0$ is observed for Na-, Li- and H-mica systems.

	Na-Mica		Li-Mica		H-Mica	
No	d	$\Delta\Omega^{ex}$	d	$\Delta\Omega^{ex}$	d	$\Delta\Omega^{ex}$
1	4.5	39.65	4.6	52.706		
2	**5.9**	**-15.3**	5.5	12.43	5.8	5
3	6.95	-2.7	5.75	12.55	6.3	7.95
4	7.5	-4.1	6.85	-1.145	7.27	2.75
5	7.9	-3.92	7.7	0.161	8	3.65
6	8.5	-4.38	**8.85**	**-3.97**	9.8	0.157
7	11.5	-1	10.3	-1.3	11	0.85
8	$\geq$ 13.5	0	11.65	-2.06	**13.5**	**-0.279**
9			$\geq$ 18	0	$\geq$ 15	0

Odd and Even numbers represent maxima and minima positions, respectively.
Reported $\Delta\Omega^{ex}$ is normalized by thermal energy (k_BT) and area of the surface (A_{xy}), thus it has units of nm.$^{-2}$
Numbers in bold indicate the d_{min} at which global minimum in $\Delta\Omega^{ex}$ is observed.

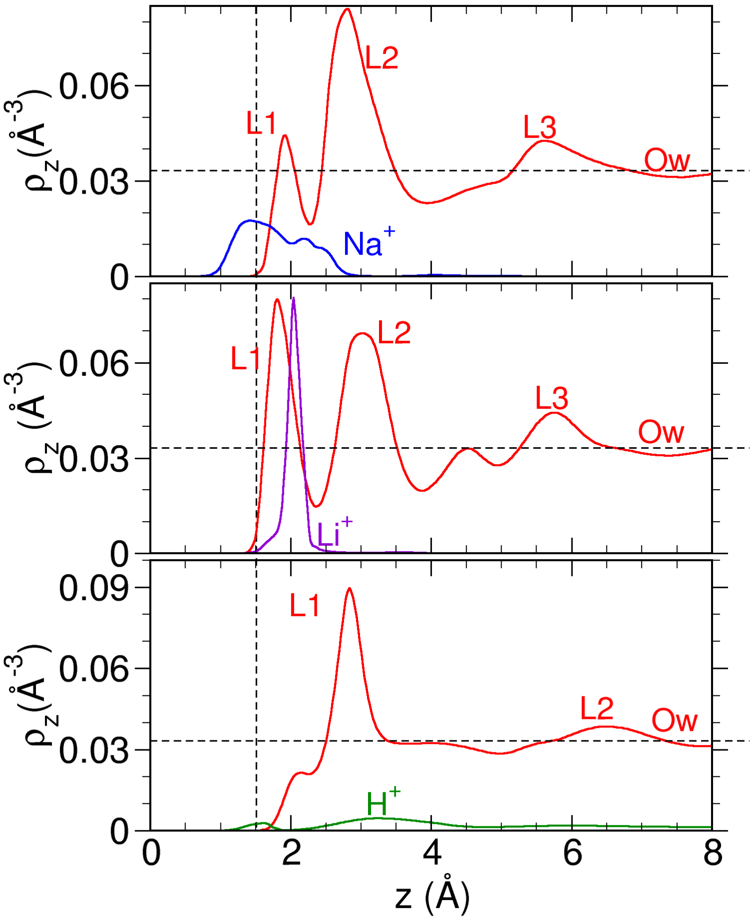

Figure S3: Density distribution of water (oxygen atom, red solid line) and Na^+, Li^+, and H^+ ion in respective mica system. The horizontal dashed line represent the bulk value of water density, whereas the vertical dashed line indicates the excluded zone of $\zeta = 1.5$ Å adjacent to mica surface.

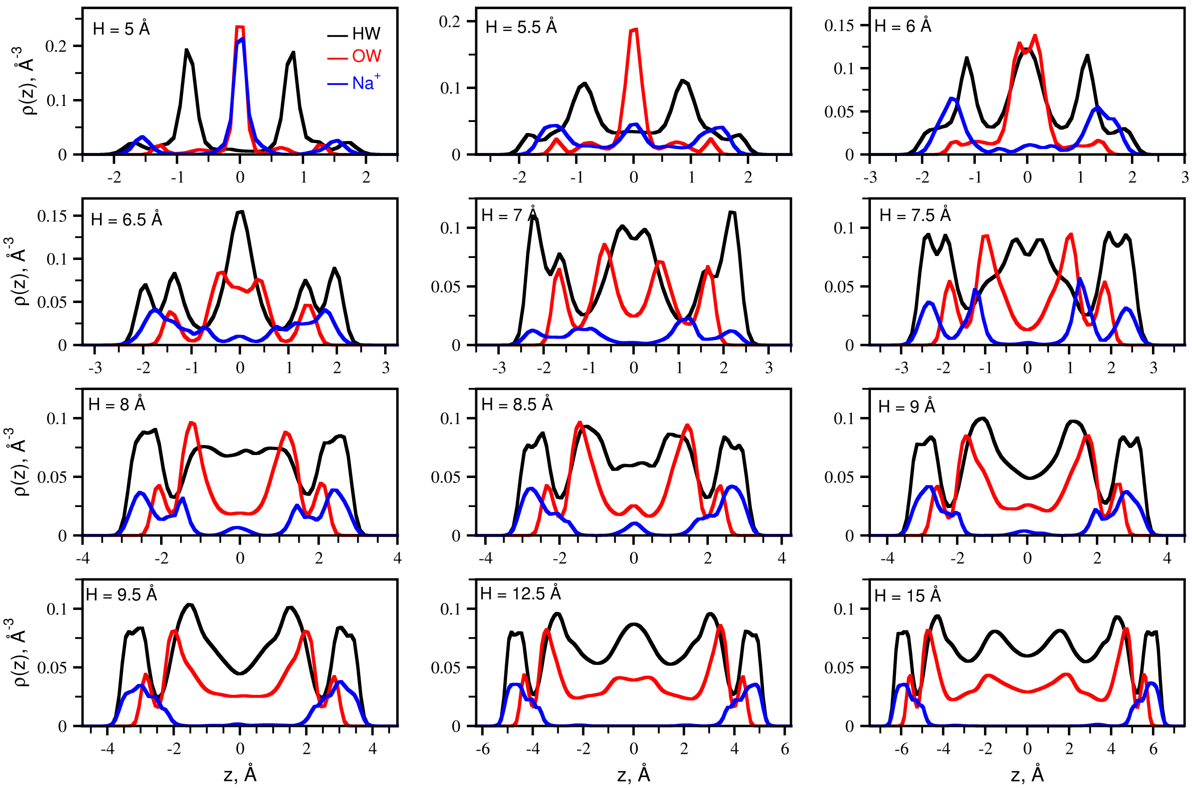

Figure S4: Density distribution of water oxygen (Ow, red solid line), hydrogen (Hw, black solid line) and cation (blue line) in Na-mica pores at various pore widths, H.

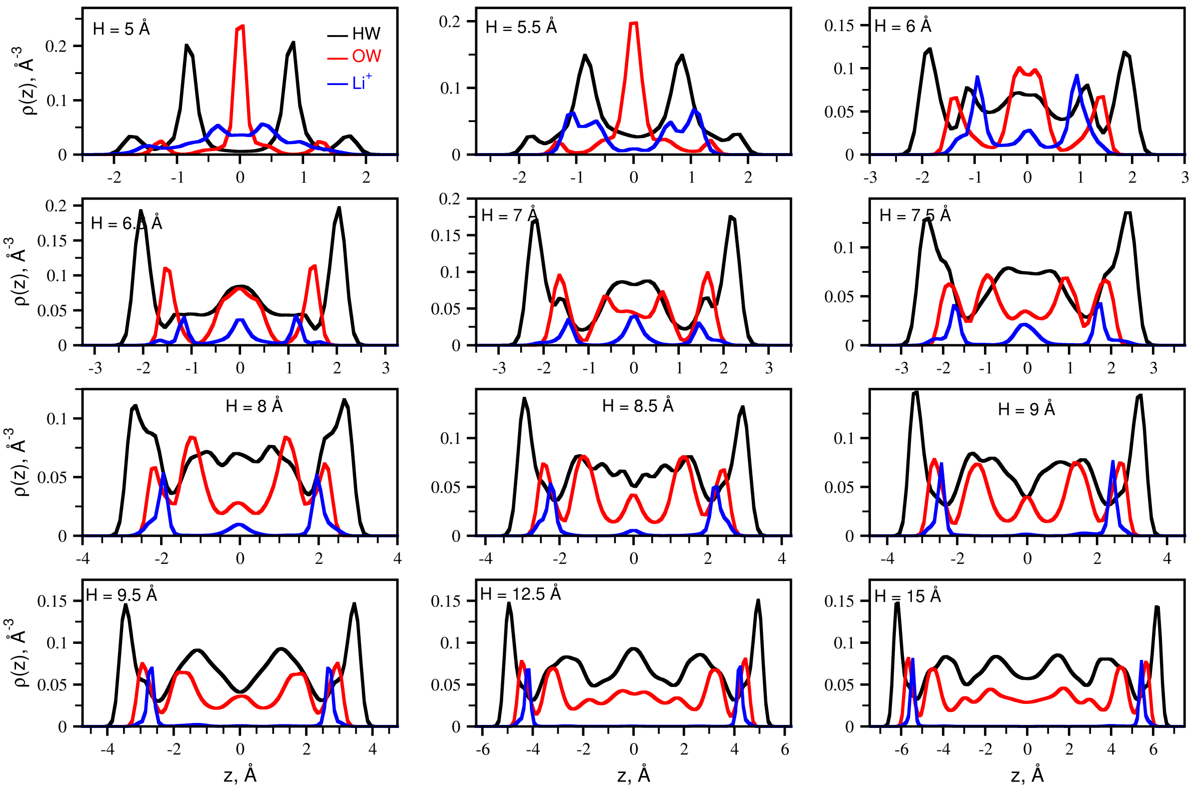

Figure S5: Density distribution of water oxygen (Ow, red solid line), hydrogen (Hw, black solid line) and cation (blue line) in Li-mica pores at various pore widths, H.

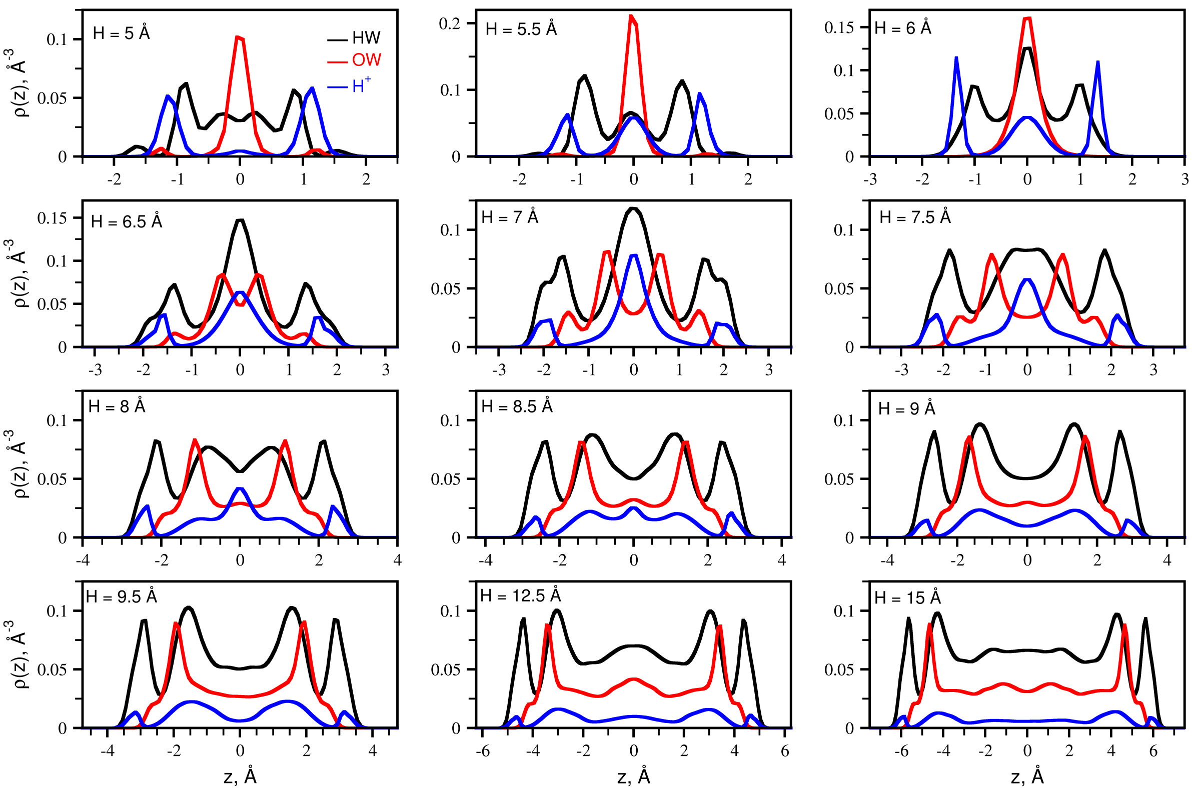

Figure S6: Density distribution of water oxygen (Ow, red solid line), hydrogen (Hw, black solid line) and cation (blue line) in H-mica pores at various pore widths, H.
