# Supplementary material for: Cation hydration by confined water and framework-atoms have crucial role on thermodynamics of clay swell﻿ing
Source: Sci Rep. 2022 Oct 24;12:17810. doi: 10.1038/s41598-022-21349-3 (PMC9592624; doi:10.1038/s41598-022-21349-3)
Supplement: Supplementary file 2 — Supplementary Information. [file 41598_2022_21349_MOESM2_ESM.docx]

Supporting Information contains additional information on i) forcefield parameters, ii) structure of water adjacent to single M-mica surface, and iii) density of water and ions in various pores of Na-, Li- and H-mica systems.
